# Comparative study of PRPH2 D2 loop mutants reveals divergent disease mechanism in rods and cones

**DOI:** 10.1007/s00018-023-04851-3

**Published:** 2023-07-19

**Authors:** Larissa Ikelle, Mustafa Makia, Tylor Lewis, Ryan Crane, Mashal Kakakhel, Shannon M. Conley, James R. Birtley, Vadim Y. Arshavsky, Muayyad R. Al-Ubaidi, Muna I. Naash

**Affiliations:** 1 Department of Biomedical Engineering, University of Houston, 3517 Cullen Blvd. Room 2027, Houston, TX 77204-5060 USA; 2 Department of Ophthalmology, Duke University Medical Center, Durham, NC USA; 3 Department of Cell Biology, University of Oklahoma Health Sciences Center, Oklahoma City, OK 73104 USA; 4 Epsilogen Ltd, Hammersmith, London, W6 9RH UK

**Keywords:** Peripherin-2, Tetraspanin, ROM1, Retinal degeneration, Retinitis pigmentosa, Pattern dystrophy

## Abstract

Mutations in the photoreceptor-specific tetraspanin gene peripherin-2 (*PRPH2*) lead to widely varying forms of retinal degeneration ranging from retinitis pigmentosa to macular dystrophy. Both inter- and intra-familial phenotypic heterogeneity has led to much interest in uncovering the complex pathogenic mechanisms of *PRPH2*-associated disease. Majority of disease-causing mutations in PRPH2 reside in the second intradiscal loop, wherein seven cysteines control protein folding and oligomerization. Here, we utilize knockin models to evaluate the role of three D2 loop cysteine mutants (Y141C, C213Y and C150S), alone or in combination. We elucidated how these mutations affect PRPH2 properties, including oligomerization and subcellular localization, and contribute to disease processes. Results from our structural, functional and molecular studies revealed that, in contrast to our understanding from prior investigations, rods are highly affected by PRPH2 mutations interfering with oligomerization and not merely by the haploinsufficiency associated with these mutations. On the other hand, cones are less affected by the toxicity of the mutant protein and significantly reduced protein levels, suggesting that knockdown therapeutic strategies may sustain cone functionality for a longer period. This observation provides useful data to guide and simplify the current development of effective therapeutic approaches for *PRPH2*-associated diseases that combine knockdown with high levels of gene supplementation needed to generate prolonged rod improvement.

**Supplementary Information:**

The online version contains supplementary material available at 10.1007/s00018-023-04851-3.

## Introduction

Peripherin-2 (PRPH2) (also known as retinal degeneration slow, RDS) is a 346-amino acid glycoprotein essential for the formation and maintenance of the disc rim region in rod and cone photoreceptor outer segments [1, 2]. Many inherited retinal diseases are linked to the more than 200 known pathogenic mutations in *PRPH2* [3, 4]. *PRPH2*-associated diseases are largely autosomal dominant [5], though digenic forms exist [6, 7], and clinical manifestations of *PRPH2* mutations can vary widely from retinitis pigmentosa to macular dystrophy [8–22]. In addition, *PRPH2*-associated diseases often exhibit phenotypic heterogeneity with disease presentation, progression, and onset often differing significantly among patients with identical mutations [7, 23]. As a result of this complexity in disease presentation, research dedicated to understanding how the structure and function of the PRPH2 protein contribute to disease pathogenesis has been of utmost importance.

PRPH2 is a member of the tetraspanin family of proteins [24], with four characteristic membrane spanning domains and a small and a large extracellular loop (called D1 and D2, respectively) [25–28]. The PRPH2 D2 loop houses seven cysteines, six of which form intramolecular disulfide bonds critical for proper D2 loop conformation [29]. The seventh cysteine (C150) mediates intermolecular associations with other PRPH2 molecules [29] as well as with the non-glycosylated PRPH2-homologue retinal outer segment membrane protein 1 (ROM1) [30, 31] (The PRPH2 structure is depicted in Fig S1). Together, PRPH2 and ROM1 associate in the inner segment to form non-covalent homo- and hetero-tetramers [32]. After trafficking to the outer segment, larger oligomers are assembled via covalent, disulfide linkages to establish and reinforce the rim domain of the disc [32–35]. In the absence of PRPH2 (i.e. in the naturally occurring null mutant known as *rd2* and now referred to as *Prph2*^*−/−*^ or *rds*^*−/−*^), rod and cone outer segments fail to form, there is no phototransduction, and photoreceptors degenerate [2, 36].

The proper formation and assembly of PRPH2/ROM1 complexes is essential for the initiation of disc biogenesis [36, 37] and for normal disc morphology, structure, and function [31, 34, 38, 39]. Examination of a model in which PRPH2 complexes cannot form intermolecular disulfide linkages (*Prph2*^*C150S*^) [40] demonstrated that proper assembly of covalent higher-order oligomers is critical to the architecture of the disc [31, 38, 40–42].

Early work exploring the mechanisms of *PRPH2-*associated disease using the *Prph2*^+/−^ model demonstrated that the PRPH2 level is critical for maintaining photoreceptor health and that PRPH2 haploinsufficiency leads to retinal degeneration, first targeting rods and later cones [43, 44]. Subsequent data from multiple transgenic and knockin mouse models have broadly suggested that haploinsufficiency (and thus loss-of-function mutations) primarily affects rods while mutations that lead to abnormalities in PRPH2 oligomerization have a greater effect in cones [45–47]. However, it is becoming increasingly clear, based on data from both patients and animal models, that this paradigm is overly simplistic.

To further expand upon our understanding of the complex pathomechanisms governing *PRPH2-*associated diseases, we investigated the functional, structural, and biochemical outcomes of mutations targeting critical D2 loop cysteines, given their role in disease and overall protein functionality. We employed three knockin mouse models bearing the *Y141C, C213Y* and *C150S* mutations, alone and in combination. Though these mutations target D2 loop cysteines, they have widely divergent disease phenotypes and effects on PRPH2 complex assembly. Two of these mutations, *Y141C* and *C213Y*, are of particular interest because, in patients, they are largely characterized by pattern dystrophy (although some Y141C patients present with retinitis pigmentosa) [22, 23, 48, 49], yet they have opposing effects on PRPH2 complex assembly. In mouse models, the Y141C mutant protein traffics properly but forms abnormal high-molecular-weight PRPH2/ROM1 complexes [39], while the *C213Y* mutation results in a protein that cannot traffic to the outer segment and does not properly oligomerize [50]. Together with the C150S mutant protein, which is stable and traffics properly but cannot form disulfide linked complexes, these mutant proteins, alone and in combination, provide valuable models to delineate the dominant biochemical features of each mutant protein and how these features differentially affect rods and cones.

## Results

### Mutations in PRPH2 destabilize critical D2 loop structures

Seventy percent of all known *PRPH2* disease-causing mutations reside in the D2 loop [22, 36]. It is a highly conserved domain that ranges from amino acids ~ 125 to 251. It is subdivided into two regions, conserved and variable, where the conserved domain is responsible for homomeric interactions, and the variable domain is responsible for tetraspanin specific protein–protein interactions. Considering the important functional and structural roles of this domain, we began our investigation by using tools to assess the possible effects on D2 loop structure as a result of mutating critical cysteines.

**Fig. 1:**
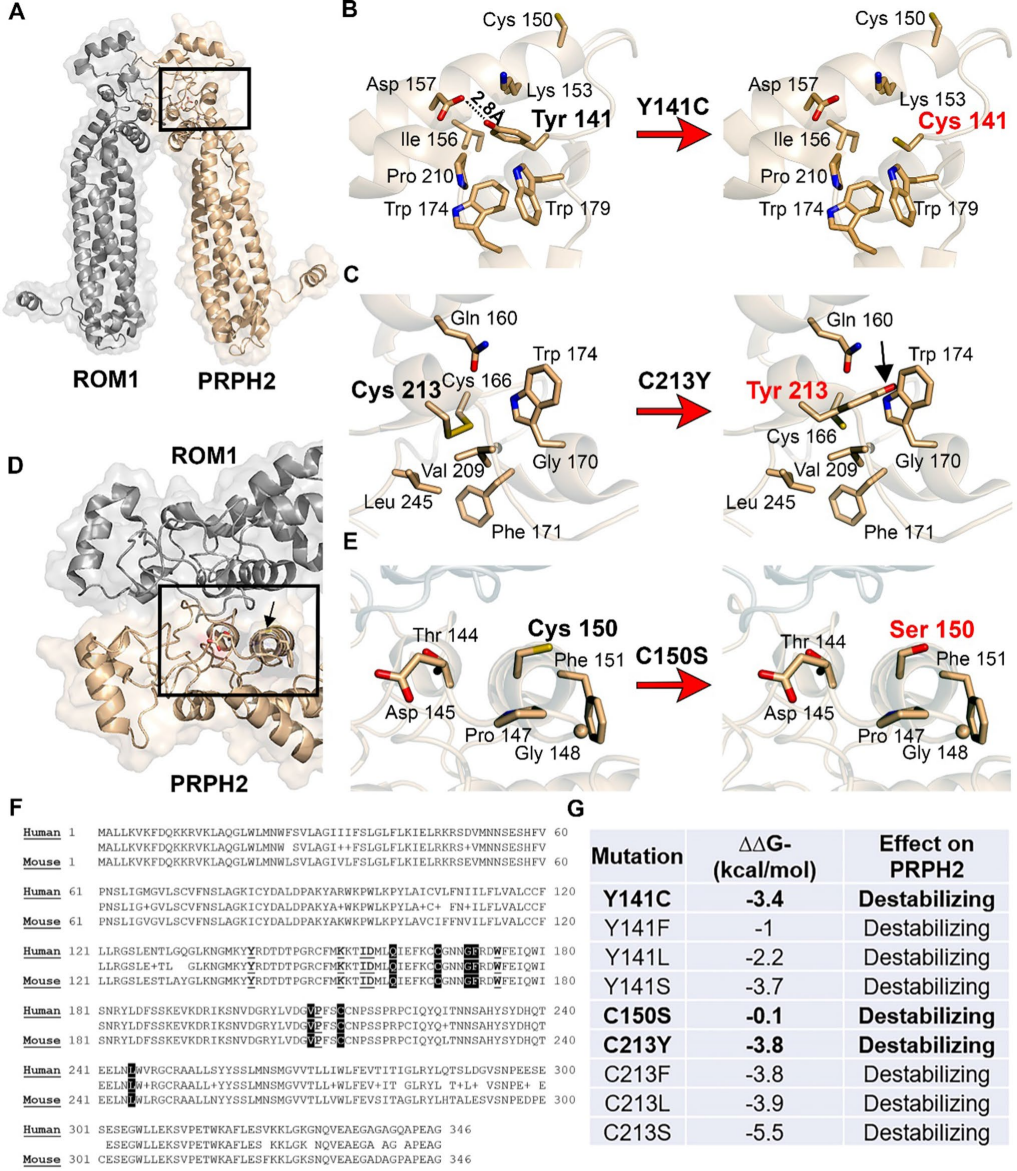
Y141C and C213Y mutations destabilize critical hydrophobic pockets of the D2 Loop. **A** Ribbon cartoon of human ROM1-PRPH2 heterodimer based on cryo-EM data (PDB: 7ZW1), generated in PyMol. **B**, **C** Image show residues Y141 and C213 and surrounding interacting amino acids. Image right of the arrow demonstrates the loss of interaction (Y141C) or clashing residues (C213Y) as a result of each mutation. Black arrow points to clashing residues. **D** Is ribbon cartoon of ROM1-PRPH2 heterodimer, showing C150 and surrounding residues on the protein’s surface. **E** Image on the right shows C150 on the surface of PRPH2 with few notable interactions. The right of the arrow, shows no significant changes as a result of the C to S mutation. **F** Pairwise sequence alignment of human (Uniprot accession P23942) and mouse (P15499) PRPH2 proteins. The middle line indicates residues in common, with a + indicating a conservative and a space a non-conservative substitution. The location of the D2 loop is shown with a grey bar below the sequence. Residues involved in the Tyr 141 hydrophobic pocket, including Lys 153, Ile 156, Asp 157, Trp 174, Trp 179 and Pro 210 are underlined and in bold. Residues associated with the Cys 213 hydrophobic pocket, including Cys 166, Gln 160, Gly 170, Phe 171, Val 209 and Leu 245 are shown in bold with white lettering. Trp 174 is common to both hydrophobic pockets and is already highlighted. **G** Table of changes in Gibbs free energy of unfolding (ΔΔG) as a result of different mutations

Examination of the contributions made by Tyr 141, Cys 150 and Cys 213 in the human PRPH2/ROM1, cryo-EM structure (PDB ID 7ZW1 [51], Fig. 1A) allowed for direct visualization of their respective locations, identification of contacting residues and assessments of their contribution to the tertiary and quaternary structures (Fig. 1B, C, E, left most panel). We expect similar changes on the mouse PRPH2 since the human and mouse PRPH2 proteins share 91% sequence identity.

Tyr 141 is buried in a hydrophobic pocket and makes a series of contacts with Lys 153, Ile 156, Trp 174, Trp 179 and Pro 210 (Fig. 1B). The terminal hydroxyl of Tyr 141 makes an intramolecular hydrogen bond with the side chain of Asp 157. This hydrophobic pocket lies on the surface of PRPH2 and side chain residues as well as on the main chain atoms to form crucial contacts at the dimerization interface with ROM1. A non-conservative mutation of Tyr 141 to the much shorter Cys (aromatic to non-aromatic amino acid would likely destabilize this pocket and negatively impact the ability to form heteromeric complexes with ROM1.

In wild-type (WT) PRPH2, Cys 213 forms an intramolecular disulfide bond with Cys 166 which is buried in a pocket lined with predominantly hydrophobic residues. Key contacts are made with the side chains of Gln 160, Gly 170, Phe 171, Trp 174, Val 209 and Leu 245 (Fig. 1C). This hydrophobic pocket lies on the surface of PRPH2, side chain residues and main chain atoms form contacts at the dimerization interface with ROM1. The non-conservative Cys 213 to Tyr mutation will be doubly impactful on the protein structure due to loss of a key disulfide bond and burying of a much larger residue into a smaller defined pocket (the black arrow points to a clash with Trp 174, as an example). This would destabilize the hydrophobic pocket and negatively affect homo- and heterooligomer formation. It is important to note that the amino acids contributing to the hydrophobic pockets which contain Tyr 141 and Cys 213 are absolutely conserved between human and mouse *PRPH2* sequences (Fig. 1F).

Residue 150 (presented as Ser in the Cryo-EM structure and Cys in the WT protein [51]) is solvent-exposed and located on the surface of PRPH2 (Fig. 1D, black arrow). It is found in a loop and the closest side chain residues are Thr 144, Asp 145, Pro 147, Gly 148, and Phe 151 (Fig. 1E). Loss of the capacity to form a disulfide bond at this location and thus the capacity for covalent homo- or hetero-pairing would negate the ability to form higher-order oligomers. The mutation to serine may still allow non-covalent oligomerization to occur under certain circumstances but these interactions would likely be less stable given the loss of the capacity to form this crucial covalent bond.

We also wanted to evaluate the degree of surface accessibility of Tyr 141, Ser 150 and Cys 213. To do this we input the PRPH2/ROM1 coordinates into the PDBePISA server (http://www.ebi.ac.uk/pdbe/pisa/) [52]. Tyr 141 is only partially accessible to solvent with an accessible surface area (ASA) of 4.98 Å^2^ (Supplementary Table 1). Ser 150 by contrast is a much smaller amino acid than tyrosine and is relatively highly solvent exposed with an ASA of 66.55 Å^2^. However, Cys 213 is completely buried within the protein and inaccessible to solvent, with an ASA of 0 Å^2^.

The impact on the thermal stability of the PRPH2, defined as the difference in the Gibbs free energy of unfolding (ΔΔG, given in kcal/mol) between the WT and the mutant proteins was calculated using the PRPH2/ROM1 Cryo-EM structure and the DDGun3D server (https://folding.biofold.org/ddgun/index.html) [53, 54] (Fig. 1G). In addition to the mutations presented in this study, we calculated the effect of substitution to various amino acid types with aromatic, branched hydrophobic or polar side chains. Amino acid substitutions classed as: negative ΔΔG kcal/mol values for destabilizers, zero values for no effects, and positive ΔΔG values for stabilizers. Interestingly, all Y141 substitutions were destabilizers with the least destabilizing effect from Y141F, which is a conservative substitution (i.e. aromatic to aromatic) (Fig. 1G). However, the most destabilizing ones are Y141C and Y141S (ΔΔG of − 3.4 and − 3.7 kcal/mol, respectively) while substitution to leucine led to an intermediate effect (− 2.2 kcal/mol).

Substitution of Cys 150 to Ser is only marginally destabilized PRPH2. This effect may have been predicted, given that Cys 150 is on the surface of the protein and solvent exposed and substitution to another residue is unlikely to clash with others.

Substitutions of Cys 213 to either Tyr, Phe, Leu or Ser were found to be very destabilizing, collectively with numbers ranging from − 3.8 to − 5.5 kcal/mol, values higher than any other mutation tested. This negative effect is likely to be a result of this residue being completely buried in a hydrophobic pocket and any substitution will clash with close residues.

To determine how the overall conformation of the D2 loop may be impacted by these mutations, the mouse PRPH2 sequence was input into the SWISS-MODEL homology modeling server, where the 3D model of both the human PRPH2-ROM1 heterodimer [51] and the crystal structure of the large extracellular loop of Tetraspanin-15 (LEL TSPAN 15, PDB ID: 7RDB [55]) were presented as the most viable templates. Predictions were evaluated using both templates. The cryo-EM structure of the heterodimer was used to understand what occurs in specific pockets of the D2 loop. However, overall structural changes required a different template that had higher resolution (2.52 Å), was in a monomeric form, and was domain-specific, given it was modeled for only the large extracellular loop, and not the quaternary structure of the protein. The WT PRPH2 prediction (Fig. S2) resulted in a structure with four characteristic helices (H1–4) consistent with the conserved tetraspanin domains, which span amino acids 125–167 and 250–263 [56]. The regions marked in magenta highlights the ROM1 (amino acids Y140–N182) binding domain while the brown region highlights the binding domain for PRPH2/PRPH2 homomeric interactions [(C165–N182), there is a significant overlap between the two domains] [57]. There is a small loop between H1 and H2 and a large loop connecting H3 and H4 consisting of many loops and turns and corresponding to the hypervariable region in the canonical tetraspanin extracellular loop 2 structure.

The Y141C substitution resulted in several changes to the overall structure. There appeared to be aberrations in the large loop (between H3 and H4) and the small loop (between H1 and H2) as well as in a region in front of H4 (Fig. S2B, top and bottom). The bottom panel of Fig. S2B–D presents structural overlays of the mutants compared to the WT, highlighting regional deviations. In the Y141C protein, the conserved tetraspanin regions remained largely unaffected except for the small loop found within the PRPH2/ROM1 binding domain which may be related to the destabilization of its hydrophobic pocket highlighted in Fig. 1B. The large loop appeared to be more unfurled with smaller loops and turns compared to the WT structure.

Different predicted structural changes occurred as a result of the C150S mutation (Fig. S2C, top and bottom). This cysteine is necessary for covalent homomeric interactions and is located in the small loop between H1 and H2. However, we observed no differences in the predicted secondary structure of the small loop. The lack of significant conformational changes to the D2 loop corroborates important biochemical properties about C150S already discussed, that C150 has a high surface accessibility (Fig. 1E and Table S1), has minimal interactions with other residues (Fig. 1E), and the substitution from Cys to Ser is conservative (Fig. 1G).

The C213Y mutation caused changes to similar regions (Fig. S2D, top and bottom) described in the Y141C. In the large loop between H3 and H4 (analogous to the other mutations) we observed structural differences from the WT loop. The loop is shorter, more compact, with obvious smaller loops within the larger one. Finally, there were no significant differences predicted towards the C-terminal end, as observed in the first two mutations. To view the effects of the mutations on the structure of the D2 loop from multiple perspectives, video files of the WT and each mutation (*Y141C, C150S, C213Y)* are included in movies 1–4, respectively.

Ultimately, in all the mutants, conformational differences are mostly located in the large loop between H3 and H4, which is critical to the overall protein structure. This loop is within the hypervariable region of tetraspanin extracellular loops and is thought to be a region critical for specific protein function [56]. Furthermore, both *C213Y* and *Y141C* are highly destabilizing mutations, impacting the region where covalent intermolecular linkages form and are found within the ROM1/PRPH2 binding domain. This correlates with changes to intermolecular binding of these two mutants, though the effects of the two mutations on PRPH2 complex assembly are not the same. The *Y141C* mutation causes aggregation of mutant PRPH2 [39] while *C213Y* prevents homomeric associations and ROM1 binding [50]. Although the outcome of the *C213Y* mutation in patients is the most devastating [48, 58], the structural deviations from the WT did not appear drastic. Visualizations of the Cys 213 to Tyr substitution in its pocket indicated severe disruptions to interaction with other residues, which would ultimately inhibit proper folding, and thus highlights the limitation of predictive tools. Nevertheless, C213 is part of a conserved motif, PxxCC (found in over 50% of tetraspanins) [24, 59–62], where mutations are known to result in severe phenotypes [63, 64]. Collectively, these analyses do provide some structural evidence for the biochemical behaviors of mutant PRPH2, which are further elucidated in subsequent sections.

### Cone photoreceptors show functional resilience in compound mutant retinas

Using our previously characterized PRPH2 knockin lines *Prph2*^*Y141C/Y141C*^, *Prph2*^*C150S/C150S*^, and *Prph2*^*C213Y/C213Y*^ (hereafter abbreviated *Y141C/Y141C*, *C150S/C150S*, and *C213Y/C213Y*), we generated compound mutant animals. These *Prph2*^*Y141C/C150S*^*, Prph2*^*Y141C/C213Y*^, and *Prph2*^*C150S/C213Y*^ (abbreviated *Y141C/C150S*, *Y141C/C213Y*, and *C150S/C213Y*) animals each carry two different mutant alleles with varied effects on PRPH2 complex assembly and trafficking to the outer segment. We evaluated differences among these six groups with additional reference to heterozygous animals carrying one mutant and one wild-type (WT) allele (*Y141C*/+, *C150S*/+, and *C213Y*/+), since they represent the situation in patients (with the exception of *C150S*/+, which is not a clinically identified mutation) and enable comparisons between the effects of WT and mutant alleles. Our prior work showed that the *C213Y* mutation had the most severe effects on retinal structure and function with the *Y141C* and *C150S* mutations eliciting slightly milder effects.

We performed full-field electroretinography (ERG) coupled with histologic assessment of retinal degeneration in compound mutant animals and controls at postnatal day (P) 30 and P90. Results from PRPH2 WT, heterozygous (*Prph2*^+/−^), and homozygous null *Prph2*^*−/−*^) animals at P30 and P90 were as expected based on previous findings (Fig. S3A, B), with *Prph2*^+/−^ animals exhibiting significant rod-dominant haploinsufficiency.

**Fig. 2:**
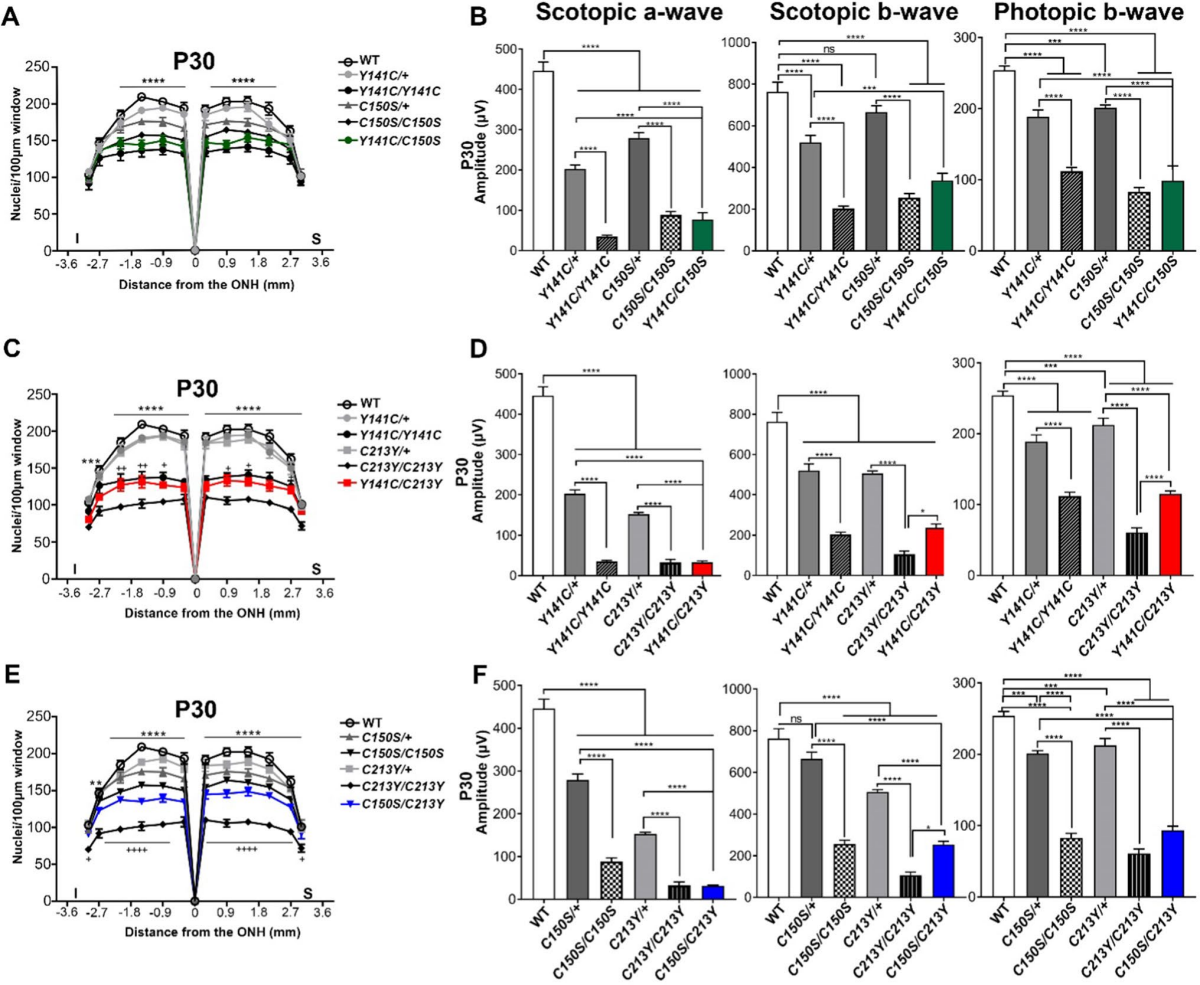
*Prph2* mutants are more deleterious to rod than to cone photoreceptors. **A**, **C**, **E** Morphometric analyses of H&E stained images were performed on retinal sections of the indicated genotypes at P30. Retinas were cut along the superior-inferior axis, and nuclei in the outer nuclear layer were counted in 100 µm windows. **B**, **D**, **F** Full-field ERGs were recorded and scotopic a-, b-, and photopic b-wave amplitudes are presented as the mean ± SEM. White, grey, and black and white patterns bars, represent values for WT, heterozygous and homozygous, respectively. Colored bars represent the values of responses from compound animals exclusively. N = 7–13 animals per group. One-way ANOVA (ERG) or two-way ANOVA (morphometry) followed by Tukey–Kramer’s post-hoc testing were used to determine statistical significance. For morphometric data, * indicate comparisons between WT and the compound mutant (**A**, **C**, **E**), + indicated comparison between the compound mutant and *Y141C/Y141C* (**C**) or between the compound and *C213Y* (**E**). All significant pairwise comparisons are marked for: (1) pairs that share at least one allele (e.g. *C150S*/+ vs. *C150S/Y141C*), (2) for homozygous animals and their associated compound lines (e.g. *Y141C/Y141C* vs. *C150S/C150S* and *Y141C/Y141C* vs. *Y141C/C150S*), and (3) for pairwise comparisons with WT. Other pairwise comparisons are not marked as they are not scientifically meaningful (e.g. *C150S*/+ vs. *Y141C/C213Y*). * is *P* ≤ 0.05, ** is *P* ≤ 0.01, *** is *P* ≤ 0.001, **** is *P* ≤ 0.0001, + is *P* ≤ 0.05, ++ is *P* ≤ 0.01, +++ is *P* ≤ 0.001, and ++++ is *P* ≤ 0.0001

At P30, the *Y141C/C150S* compound mutant animals already exhibited significant retinal degeneration (fewer nuclei in the outer nuclear layer, ONL) compared to WT and was similar to both the homozygous *Y141C/Y141C* and *C150S/C150S* lines (Fig. 2A and Fig. S5A). Scotopic a-wave responses (an indicator of rod photoreceptor function) in the *Y141C/C150S* mutant retina were low, about 19% of the WT (Fig. 2B, left). Responses from second-order neurons (scotopic b-wave), and cones (photopic a and b-wave) in the *Y141C/C150S* mutant retina were not as severely impacted as the scotopic a-wave (Fig. S4 and Fig. 2B, middle and right) but were still reduced to about 50%, 41%, and 30% of the WT, respectively. These defects were worsened at P90 (Fig. S6A and B) but largely exhibited similar patterns to P30.

One of our goals was to understand which mutation in each compound mutant had the “dominant” effect, so we compared the findings in the *Y141C/C150S* line to those in the *Y141C/Y141C* and *C150S/C150S* lines. At P30 (Fig. 2B), there were no significant differences in scotopic or photopic b ERG responses between these three groups, though the *Y141C/Y141C* animals had the worst rod response while cones performed the worst in *C150S/C150S* animals consistent with previous findings [39, 40]. There was a statistically significant increase in photopic a responses of *Y141C/C150S* over *C150S/C150S* retinas (Fig S4A). However, at P90 (Fig. S6B), photopic b-waves in the *Y141C/C150S* were significantly higher than the *C150S/C150S*, suggesting that the *Y141C* allele can slow the loss of cone function associated with the C150S mutation. In a similar vein, scotopic b-wave responses in the *Y141C/C150S* were significantly higher than in the *Y141C/Y141C* (though a-waves were unaffected), suggesting that the presence of the *C150S* allele in this compound mutant had a less severe impact on rod function compared to the *Y141C/Y141C* scenario (Fig. S6B). This small degree of improvement is also observed morphometrically where photoreceptor degeneration was slightly less severe in the peripheral retina in the *Y141C/C150S* compared to the *Y141C/Y141C* (Fig. S6A, + symbols).

We next examined the *Y141C/C213Y* genotype. As with the *Y141C/C150S*, the *Y141C/C213Y* exhibited significant structural and functional defects when compared to WT at both P30 and P90 (Fig. 2C, D, Fig. S6C, D, Fig. S5A and B). *Y141C/C213Y* scotopic a-wave values were equivalent to both the *C213Y/C213Y* and *Y141C/Y141C* responses at P30 and P90 (Fig. 2D, left, Fig. S6D, left). Though rod response in the *Y141C/C213Y* retinas were miniscule (Fig. 2D, left); cone responses were more robust at P30 and were significantly better than those in the *C213Y/C213Y* (Fig. 2D, right)*.* Cone responses resembled those in the *Y141C/Y141C* and *Y141C/C150S*, further supporting our observation that the *Y141C* is a less deleterious mutant allele than C150S or C213Y on cone function, and thus replacing one allele of *C150S* or *C213Y* with *Y141C* improved cone function compared to mutants with only *C150S* or *C213Y* alleles (e.g., *C213Y/C213Y* or *C150S/C150S*).

This tendency of the *Y141C/C213Y* to mimic the *Y141C/Y141C* rather than the *C213Y/C213Y* held true when looking at the degree of retinal degeneration. At both P30 and P90, the level of degeneration was not different between the *Y141C/C213Y* and the *Y141C/Y141C*. The *C213Y* mutation was especially detrimental; at P30 there was significantly more degeneration in the *C213Y/C213Y* than in the *Y141C/C213Y* or in the *Prph2*^*−/−*^ (Fig. 2C and see + symbols Fig. S3D).

As with the other compound mutant retinas, scotopic a- and b-responses were severely reduced in the *C150S/C213Y* at both P30 and P90 (Fig. 2F and S6F). Photopic a and b responses were also severely reduced at P30 and P90 (photopic b responses only), and were not different between the *C213Y/C213Y*, *C150S/C213Y*, and *C150S/C150S* (Fig. 2F, right, Fig. S4C, Fig. S6F, right). Interestingly, scotopic b responses were significantly improved in the *C150S/C213Y* vs. the *C213Y/C213Y* at P30 (Fig. 2F, middle). This improvement to rod health over the *C213Y/C213Y* was also observed in P30 morphometry, where there were significantly more photoreceptor nuclei in the *C150S/C213Y* (Fig. 2E). However, this increase in scotopic b responses conferred by the *C150S* allele was lost by P90 (Fig. S6F).

Combined, these data suggest that: (1) rod function is very poor in all homozygous and compound mutant retinas; (2) cone function in compound mutant retinas is preserved better than rod function and that the *Y141C* allele, in particular, can improve cone function compared to retinas that contain only the *C150S* or *C213Y* alleles. However, heterozygous animals carrying one *PRPH2* WT allele and one mutant allele (*Y141C*/+,* C150S/*+, and *C213Y*/+) had better rod and cone function and less degeneration than their homozygous or compound mutant animals. This observation clearly indicates that regardless of the small benefit conferred by substituting a less detrimental mutant allele for a more detrimental one, the improvement is never as good as that provided by WT PRPH2. As one example, photopic ERG responses in the *Y141C/C213Y* are significantly better than in the *C213Y/C213Y* but are not as good as *C213Y*/+ (Fig. 2D left, and Fig. S4B).

### The *C150S* allele causes reduced cone number and mislocalization of short-wavelength cone opsin

**Fig. 3:**
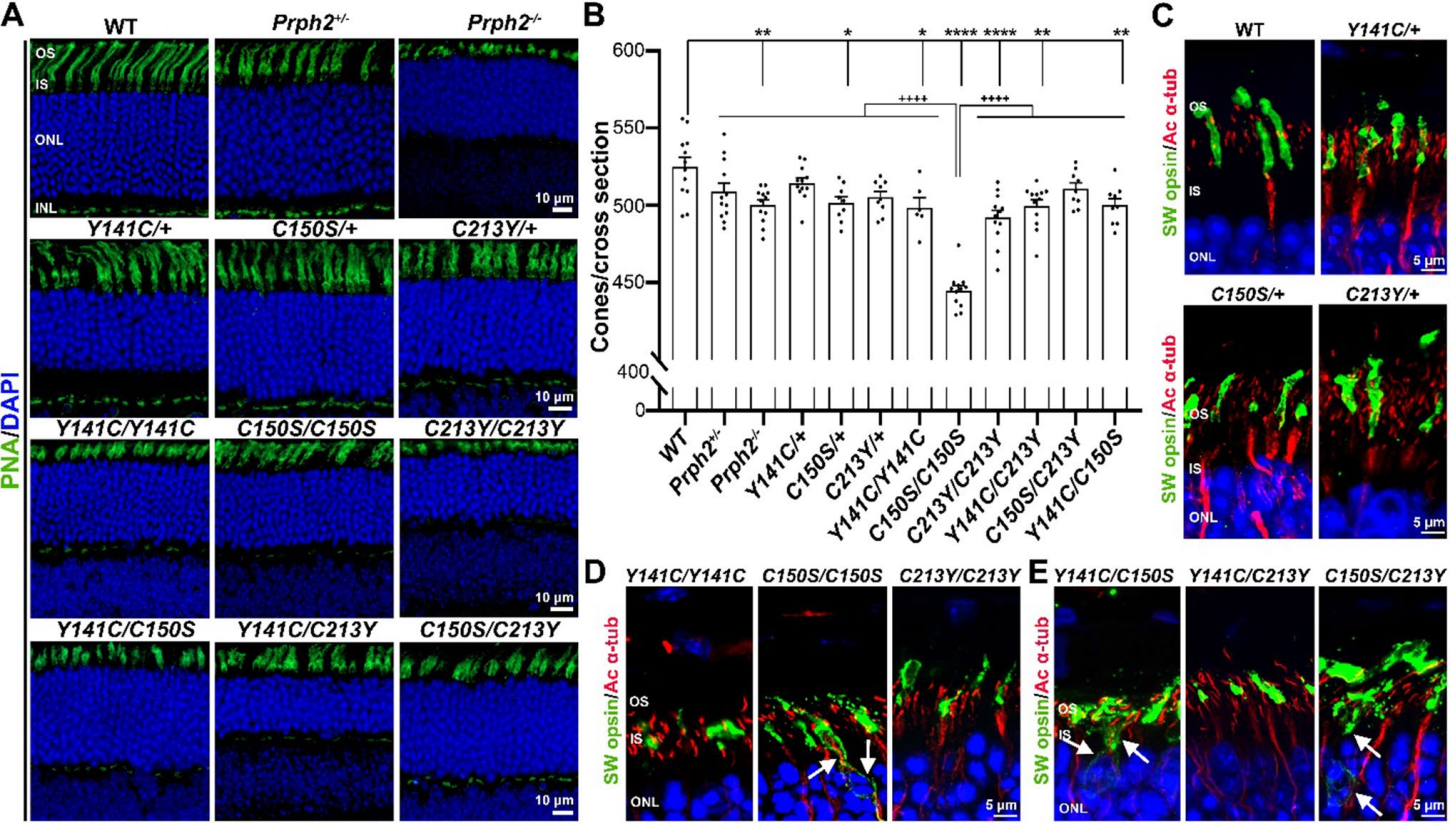
The effects of *Prph2* mutants on cone photoreceptors. **A** Cross sections of P30 retinas were stained with peanut agglutinin (PNA-green) to label cone photoreceptors. Images were captured as ×40 z-stacks. Scale bar is 10 µm. **B** PNA-labeled cone photoreceptors were counted in each cross section (plotted are mean ± SEM). Significance was determined using one-way ANOVA followed by Tukey–Kramer’s post hoc comparison analyses. Four cross sections were counted per retina, and 3 retinas from independent animals were evaluated per genotype. * is *P* ≤ 0.05, ** is *P* ≤ 0.01 and **** is *P* ≤ 0.0001. **C–E** P30 retinal sections were stained for cone opsin (SW opsin, green) and acetylated α-tubulin (red). Nuclei were counterstained with DAPI. Arrows highlight regions of SW opsin mislocalization. Scale bar is 5 µm. *OS* outer segment, *IS* inner segment, *ONL* outer nuclear layer, *INL* inner nuclear layer

Overall, our ERG studies demonstrated that cones largely proved more resilient to the presence of PRPH2 mutants (including compound mutants) than rods. To determine whether this phenomenon reflected better survival of cones vs. rods, P30 retinas were stained with the cone marker peanut agglutinin (PNA, green Fig. 3A) and the number of cones was counted in cross sections (Fig. 3B). Most of the homozygous and compound mutant lines exhibited cone numbers that were statistically significantly decreased from the number of cones in the WT, but to a much smaller degree than that observed in rod numbers. For example, the cone count in the *Y141C/C213Y* decreased by 5% compared to WT, while rod numbers in the central retina (i.e. total ONL count) were decreased by 38% compared to WT at the same age, reflecting a general resiliency of cones. However, there was one striking exception, the *C150S/C150S*. The number of cones in *C150S/C150S* retinas decreased by 15% from the WT and was also significantly decreased from other groups. The finding that the number of cones was significantly better in the *Y141C/C150S* vs. *C150S/C150S* is consistent with the ERG findings that cone function was preserved in the *Y141C/C150S* retina compared to the *C150S/C150S* retina (though not statistically significant). This paradigm does not hold true for the *C150S/C213Y*; however, cone ERG responses in the *C150S/C213Y* and the *C150S/C150S* were similar but the *C150S/C213Y* had significantly less cone loss than the *C150S/C150S* (Fig. 3B). Furthermore, though we observe that the number of cones in *C150S/C150S* retinas was significantly decreased compared to the other mutants, there were no significant differences in cone ERG functions between *C150S/C150S* retinas and any other homozygous at P30.

Protein mislocalization is a hallmark of cellular dysregulation, and cone opsin mislocalization often accompanies or precedes cone degeneration [65, 66]. To evaluate whether opsin may be mislocalized in mutant cones, retinal sections were co-labeled for short-wavelength cone opsin (SW opsin) and acetylated α-tubulin (ac α-tubulin), a marker enriched in the connecting cilia and axoneme at the base of the outer segment (Fig. 3C–E). Consistent with the cone-count results, *C150S/C150S* retinas exhibited mislocalization of SW opsin (arrow Fig. 3D). Neither of the compound mutant lines containing C150S were able to completely attenuate the mislocalization of SW opsin caused by the *C150S* allele. There was a considerable amount of mislocalized SW opsin in *Y141C/C150S* and *C150S/C213Y* retinas (arrows, Fig. 3E). The observation that cone opsin was mislocalized in compound mutant retinas containing the C150S allele suggests that cone degeneration may be incipient, even if it was not yet severe at P30.

### Compound mutants differentially affect PRPH2 protein/complex stability

**Fig. 4:**
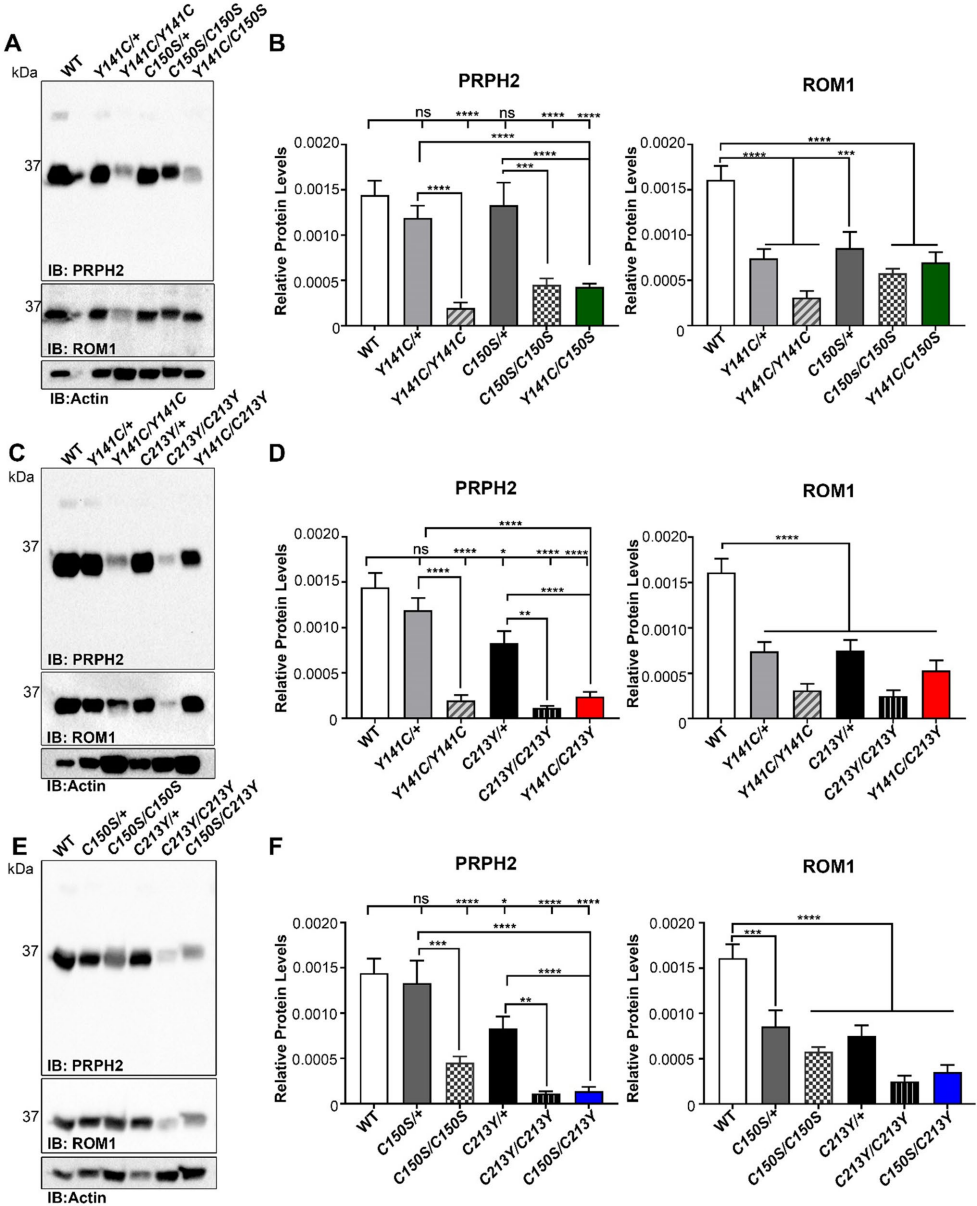
PRPH2 and ROM1 levels in cysteine-mutants. In order to assess levels of PRPH2 and ROM1, P30 WT, heterozygous and homozygous retinal extracts were subjected to assessment by reducing SDS-PAGE/immunoblotting (**A**, **C**, and **E**). Blots were probed with anti-PRPH2 (2B7) and anti-ROM1 (2H5). Quantified amounts of protein (**B**, **D**, and **F**) were determined densitometrically, normalized to β-actin, and then normalized to the total number of nuclei present in each model (determined by morphometric analysis). Values are presented as mean ± SEM. N = 4–6 samples per group. Statistical significance was determined by one-way ANOVA and Tukey–Kramer’s post hoc comparison analysis. * is *P* ≤ 0.05, ** is *P* ≤ 0.01, *** is *P* ≤ 0.001 and **** is *P* ≤ 0.0001

We next sought to explore potential biochemical and cellular mechanisms that could contribute to the improvement in cone function associated with the *Y141C* allele (in the presence of the *C213Y* or *C150S*), and the improvement in scotopic b responses associated with either the *Y141C* or *C150S* (in the presence of *C213Y*). To begin, we assessed the relative amounts of PRPH2 and its binding partner ROM1 in each model using immunoblotting (Fig. 4) and anti-PRPH2 antibodies that recognize both WT and mutant PRPH2. Values obtained from non-saturated immunoblots were normalized to actin (as a loading control) and then normalized to the total number of photoreceptor nuclei (Fig. 2 and Fig. S5). This additional layer of normalization permits the calculation of a relative level “per cell” since the significant loss of photoreceptor cells seen in Fig. 2 and Fig. S5 and previously reported [39, 40, 50] can complicate interpretation of varying protein levels. Messenger RNA levels for individual homozygous mutant *PRPH2* alleles have been previously assessed and have been shown to be equivalent to WT [39, 40, 50].

*Y141C*/+ and *C150S*/+ retinas harbored PRPH2 levels comparable to WT. This was striking since both lines exhibited significant loss of rod function, suggesting that haploinsufficiency is not the only mechanism for rod deterioration in these mutants. PRPH2 levels in *Y141C/Y141C* and *C150S/C150S* retinas were reduced compared to both WT and heterozygotes (Fig. 4A, B). *Y141C/C150S* exhibited PRPH2 protein levels comparable to the *C150S/C150S*, with mean values higher than those in the *Y141C/Y141C,* though differences were not statistically significant. The *C213Y* mutation inhibits association with ROM1 and self-oligomerization [50], and protein levels in the *C213Y*/+ and *C213Y/C213Y* were substantially reduced compared to WT (Fig. 4C, D). In the *Y141C/C213Y* retina, levels of PRPH2 were equivalent to *Y141C/Y141C* and slightly higher than the *C213Y/C213Y,* though the difference was not statistically significant. PRPH2 levels in *C150S/C213Y* retinas were similar to those in the *C213Y/C213Y* and were reduced compared to the *C150S/C150S* (reduction is not statistically significant) (Fig. 4E, F). To help provide context for interpreting the protein levels in the compound mutant retinas, we plotted the amount of PRPH2 in each homozygous retina (Fig. S7) and included black lines to divide bars at 50% to provide a visual estimate of how much protein a single mutant allele would be expected to produce. The third bar in each graph plots the “predicted” amount for each compound mutant retina (i.e. for *Y141C/C150S*, the predicted amount is equivalent to the sum of one allele of C150S and one allele of Y141C). Finally, the fourth bar plots the total amount of measured PRPH2 from the compound mutant retina. In the case of the *Y141C/C150S* and *Y141C/C213Y*, the measured level was higher than the predicted level, suggesting that the C150S can stabilize the Y141C in the *Y141C/C150S* while the Y141C can stabilize the C213Y in the *Y141C/C213Y* retina. However, this was not the case with the *C150S/C213Y* as the predicted level was higher than the measured level, suggesting that the C213Y mutant protein destabilized the C150S. The finding that the *C150S/C213Y* retina had less than the predicted amount of PRPH2 protein suggests that removing two cysteines was more destabilizing than removing one or adding one cysteine (i.e., in the *Y141C/C150S* or *Y141C/C213Y*).

It has previously been observed that levels of ROM1 are affected by the levels of mutant PRPH2 [39, 50]. Thus, it was not surprising to find that ROM1 levels in the compound retinas were reduced compared to WT (Fig. 4B, D, F). Similar patterns were observed between PRPH2 and ROM1 levels in compound mutant retinas when compared to homozygotes, and ROM1 levels correlated with reduced PRPH2 levels. ROM1 levels in both the *C213Y/C213Y* and *C150S/C213Y* were very low compared to all other genotypes evaluated, likely due to the inability of the C213Y-PRPH2 mutant to bind to ROM1.

**Fig. 5:**
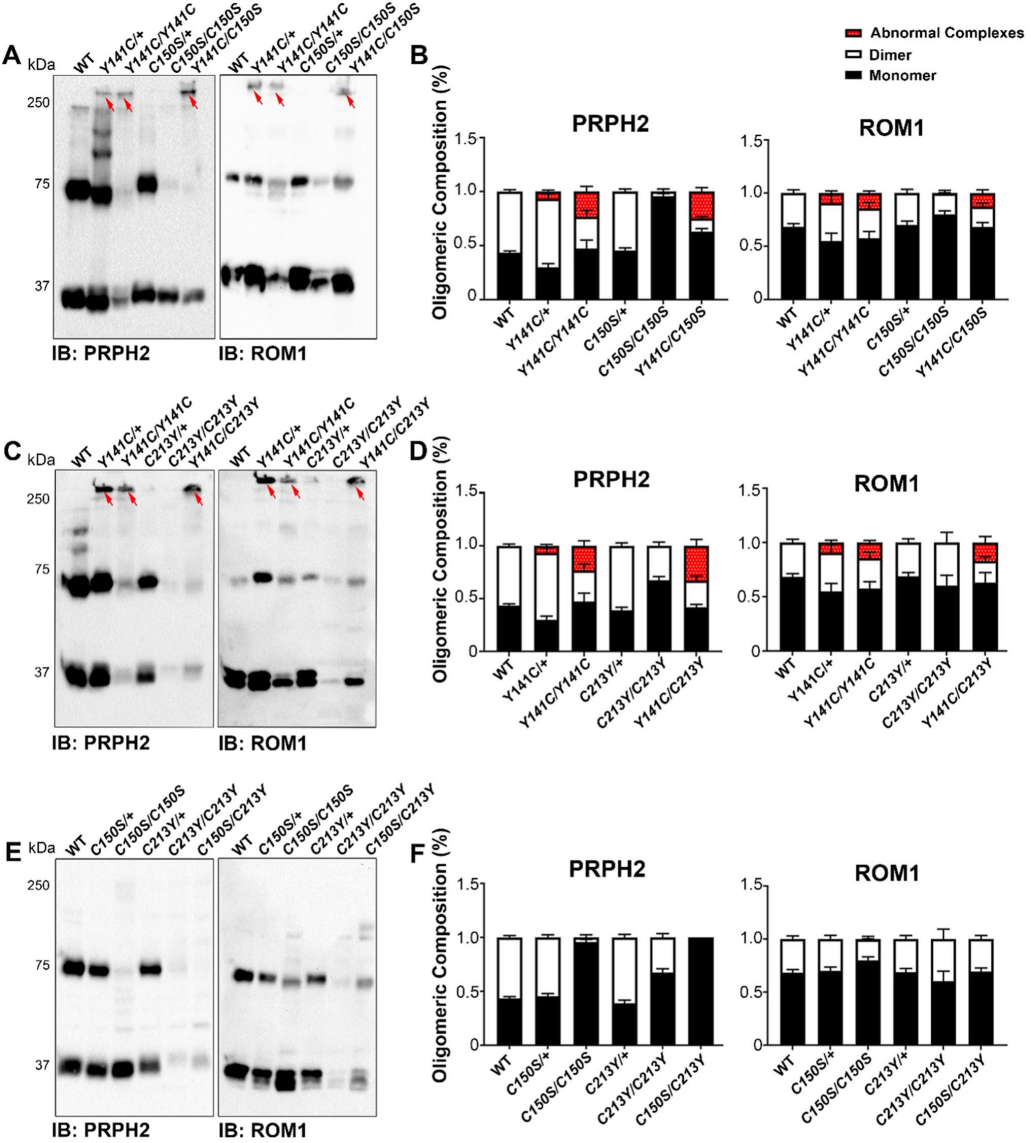
PRPH2 complex assembly in cysteine mutants. **A**, **C**, **E** To evaluate the oligomeric composition of PRPH2 and ROM1 at P30, retinas were subjected to SDS-PAGE/immunoblotting under non-reducing conditions. Blots were probed with anti-PRPH2 (2B7) or anti-ROM1 (2H5). Red arrows highlight abnormal high molecular weight complexes. **B**, **D**, **E** Bar graph display percent of total PRPH2 protein found as monomer, dimer, and abnormal high molecular weight oligomers. N = 4–6 samples per group, and values are presented as mean ± SEM. Statistical comparisons can be found in Supplementary Table 2.

PRPH2 functions as homo and as hetero-oligomer (with ROM1) assembled via both non-covalent bonds and disulfide linkages. To gain more insight into the oligomeric composition in the compound mutant retinas, non-reducing immunoblotting was performed (Fig. 5A, C, E) to distinguish between covalently- and non-covalently-linked PRPH2 and ROM1 complexes (migrating as dimers at ~ 75 kDa and monomers at ~ 37 kDa, respectively). As expected, there was no dimer in *C150S/C150S*, since C150 is responsible for the intermolecular disulfide linkages, which fail to form in the C150S. Both *Y141C*/+ and *Y141C/Y141C* exhibited the characteristic higher order aggregates (red arrows Fig. 5A, C) that have been previously observed [39], with aggregates contributing to ~ 30% of total PRPH2 in the *Y141C/Y141C* (Fig. 5B). PRPH2 in the *Y141C/C150S* retina adopted features of both the *Y141C/Y141C* and *C150S/C150S*, exhibiting a large amount of high molecular weight aggregates (like the *Y141C/Y141C*) and very little covalently-linked PRPH2 dimer (like the *C150S/C150S*, Fig. 5B). The C213Y mutant protein was present as a monomer and dimer in both the *C213Y/C213Y* and the *C213Y*/+ with no abnormal higher-order aggregates. In contrast, in the *Y141C/C213Y* compound mutant retina*,* 40% of the total PRPH2 protein existed as abnormal higher order complexes (significantly more than *Y141C*/+) with the remainder found in the dimeric form (~ 25%) and the monomeric form (~ 35%). The amount of PRPH2 found in the high molecular weight aggregates in the *Y141C/C213Y* retinas (~ 40%) was more than in the *Y141C/Y141C* (~ 30%). These two genotypes had similar total PRPH2 levels, so this increase in higher order aggregates in the *Y141C/C213Y* suggests that combining C213Y and Y141C protein leads to even more abnormal complex formation than what occurs with Y141C alone. Immunoblotting of *C150S/C213Y* retinas showed a similar dimer/monomer distribution to *C150S/C150S* retinas, without covalently-linked PRPH2. Summary of statistically significant comparisons can be found in Supplementary Table 2.

ROM1 does not participate in the largest covalently-linked PRPH2 oligomeric complexes [32], so a larger percentage of ROM1 protein existed as a monomer (arising from non-covalently linked complexes) across genotypes (Fig. 5B, D, F). Additionally, ROM1 was also present in the higher-order aggregates observed in *Y141C* models including the *Y141C/C150S* and the *Y141C/C213Y.* Interestingly, in the *C150S/C150S,* ROM1 was present in monomeric and dimeric forms, suggesting that even without covalently linked PRPH2, some ROM1 assembled into covalently-linked complexes. There was no statistically significant difference in ROM1 oligomers between genotypes.

### C213Y PRPH2 exhibits trafficking defects that are corrected by the presence of Y141C or C150S PRPH2

**Fig. 6:**
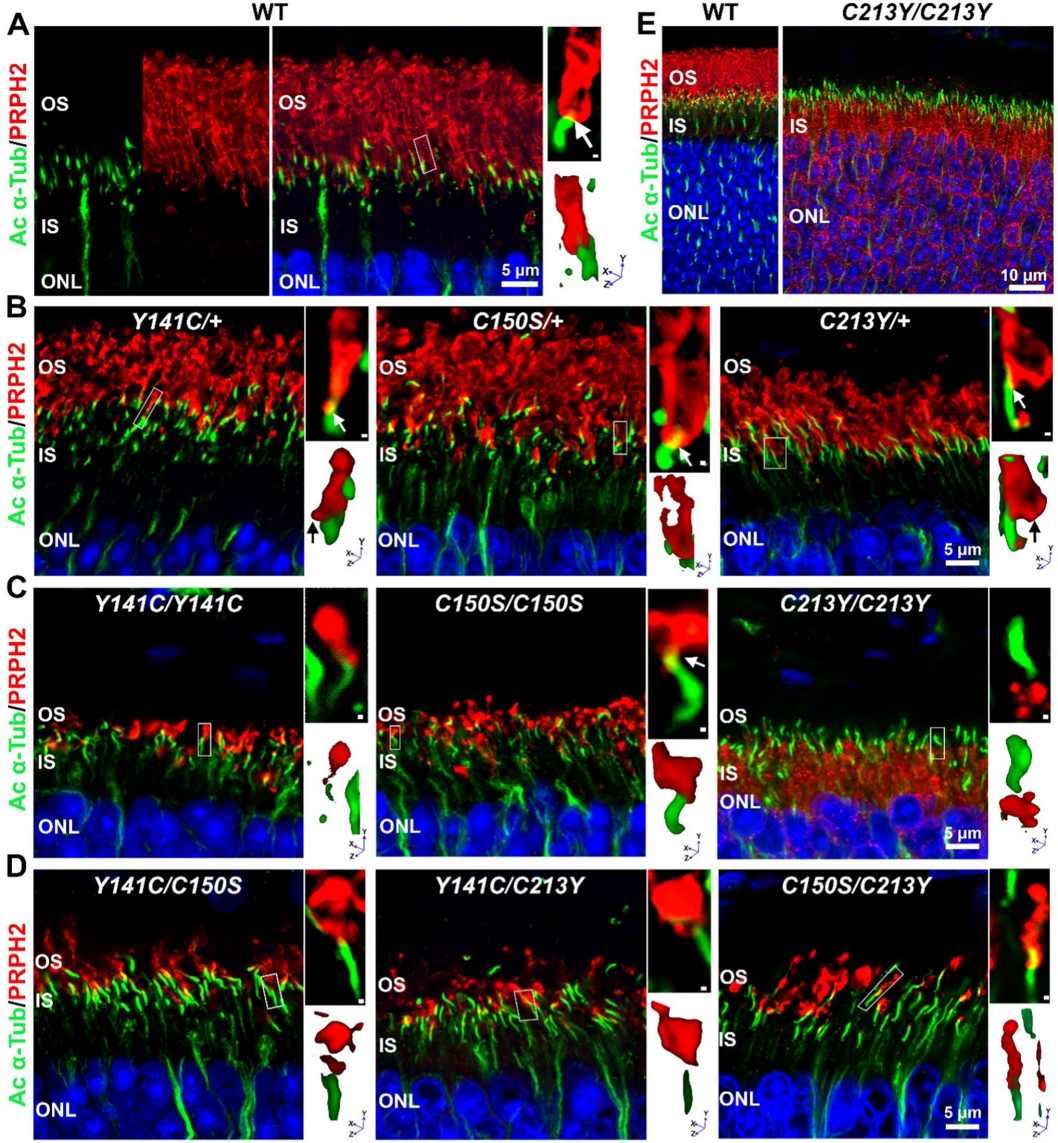
Compound mutants *Y141C/C213Y* and *C150S/C213Y* show no inner segment retention. **A–D** P30 retinal cross sections were labeled for PRPH2 (red) and acetylated α-tubulin (green). White boxes mark PRPH2-labeled photoreceptors used to generate 3D renders found in smaller panels adjacent to image. Black arrows highlight protrusions at the base of the outer segment and white arrows pointing to area of overlap between PRPH2 and acetylated α-tubulin. All images are collapsed z-stacks captured at ×63 with ×2.5 applied zoom. **E** Shown are wider fields of view from collapsed z-stacks captured at ×63. Scale bars in large panel is 5 µm, and 0.5 µm in small adjacent panels and 10 µm in panel **E**. *OS* outer segment, *IS* inner segment, *ONL* outer nuclear layer

Having evaluated the distribution of PRPH2 and ROM1 in covalent and non-covalent complexes, we also asked how these biochemical changes affected their trafficking and outer segment localization. Retinal sections were immunofluorescently labeled for PRPH2 and acetylated α-tubulin. In WT retinas, PRPH2 was in the outer segment, almost exclusively distal to the acetylated α-tubulin labeling, with only small regions of co-localization with acetylated α-tubulin at the base of the outer segment reflecting continuation of the axoneme into the outer segment (Fig. 6A, white arrow on upper right panel which is magnification of boxed area; 3D rendering is shown in lower right panels). In *Y141C*/+, *C150S*/+, and *C213Y*/+ retinas*,* PRPH2 trafficked normally to the outer segment, exhibiting a pattern similar to the WT (Fig. 6B). However, as indicated by the swollen outer segment appearance in 3D volume renders, these mutations severely affected outer segment shape, suggesting that there could be problems with disc formation.

In the *Y141C/Y141C* and *C150S/C150S*, although outer segments were extremely short, PRPH2 still trafficked to the outer segment, without accumulating in the inner segment or connecting cilium (Fig. 6C). In contrast, PRPH2 in the *C213Y/C213Y* retina was exclusively localized to the inner segment and failed to enter the connecting cilium (Fig. 6C, E). Despite both *Y141C/C213Y* and *C150S/C213Y* mutant retinas contain one allele of *C213Y,* surprisingly no inner segment retention of PRPH2 was observed. PRPH2 was properly localized distal to the connecting cilium (Fig. 6D).

**Fig. 7:**
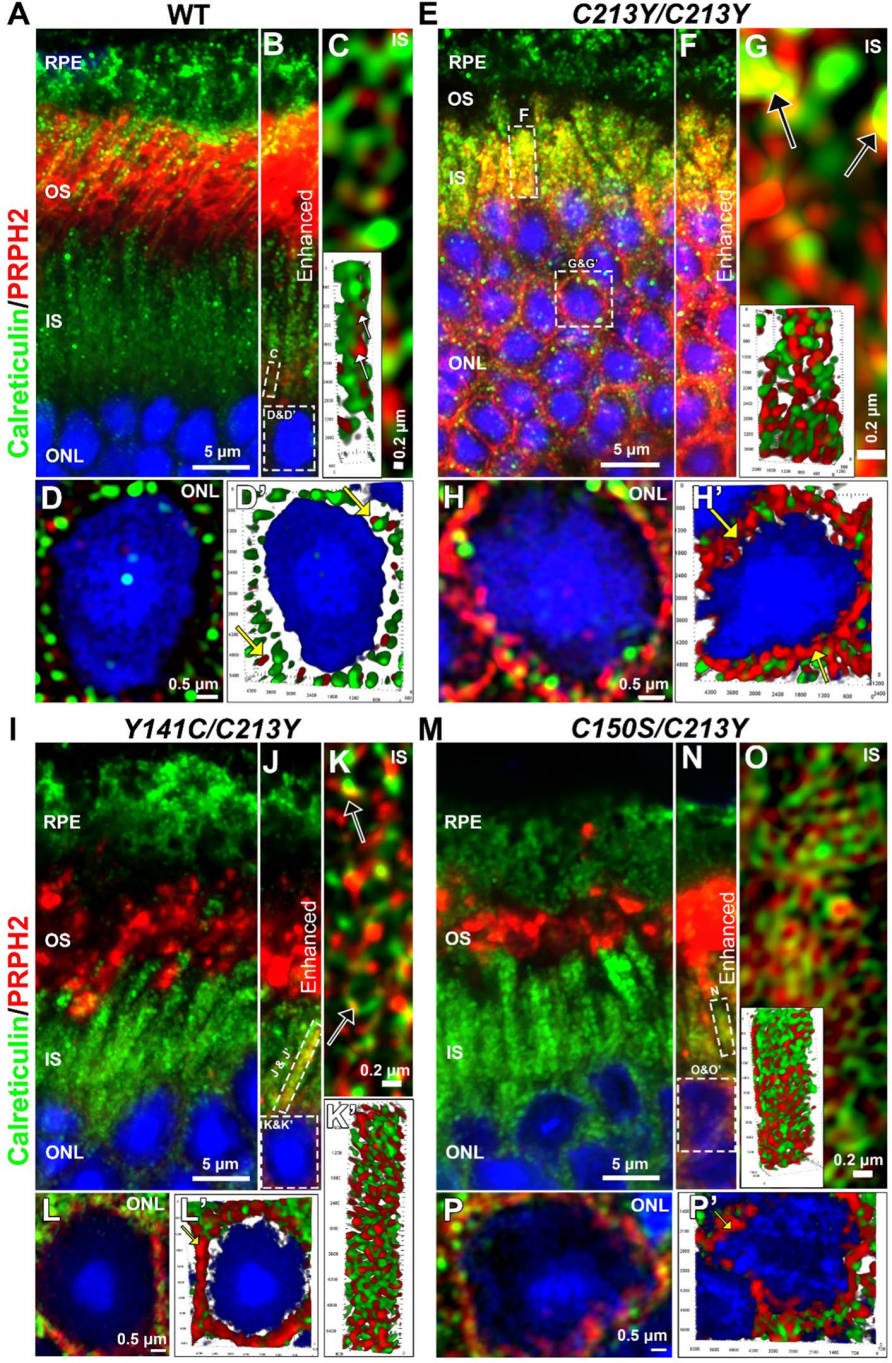
*C213Y/C213Y* retinas show PRPH2 in the ER and inner segment. P30 retinal cross sections of WT and C213Y-containing mutant retinal sections were labeled for PRPH2 (red) and the ER marker calreticulin (green). **B**, **F**, **J**, and **N** are portions of images shown in **A**, **E**, **I**, and **M** (respectively) in which the PRPH2 signal intensity was increased past saturation of the outer segment in order to visualize the small amount of PRPH2 in the inner segment. **F** is a enhance portion of the image in **E**, and was included for comparison only. **C**, **G**, **K**, **K′** and **O** are images (or 3D renders) of PRPH2 and calreticulin labeling in the inner segment region marked in the adjacent panel. Images were captured using high-resolution Airyscan confocal imaging. Black arrows point to areas of colocalization. **D**, **D′**, **H**, **H′**, **L**, **L′**, **P**, and **P′** are images of perinuclear localization of PRPH2 and calreticulin captured using Airyscan. The images were expanded from the regions marked in the panel above. 3D renders were generated from high-resolution images. Yellow arrows point to perinuclear localization of PRPH2. Scale bar is 5 µm in **A**, **E**, **I**, and **M**, 0.2 µm in **C**, **G**, **K**, and **O**, and **N** 0.5 µm in **D**, **H**, **L**, and **P**. *RPE* retinal pigment epithelium, *OS* outer segment, *IS* inner segment, *ONL* outer nuclear layer

It was not clear whether inner segment retention of C213Y-PRPH2 was the result of defects in outer segment targeting or problems in ER-exit, therefore, WT, *C213Y/C213Y, Y141C/C213Y,* and *C150S/C213Y* retinal sections were co-labeled for PRPH2 and calreticulin, an ER marker. In the WT retina, newly synthesized PRPH2 is efficiently trafficked to the outer segments (Fig. 7A). To visualize newly synthesized PRPH2 in the WT retina, image intensity was increased to levels that saturated outer segments (Fig. 7B), and punctate PRPH2 became visible in the inner segment and perinuclearly consistent with synthesis in the inner segment prior to trafficking to the outer segment (Fig. 7B–D). In WT rods, PRPH2 was present at low levels in the inner segment (white arrows in the inset of Fig. 7C) and around the nucleus (yellow arrows Fig. 7D′), but did not exhibit appreciable co-localization with calreticulin suggesting that the newly synthesized PRPH2 was quickly trafficked out of the ER. In contrast, in the *C213Y/C213Y* retina, PRPH2 immunoreactivity was observable in the inner segment without increasing the image intensity (enhanced imaged was included for comparison (Fig. 7F), reflecting the abnormal accumulation of C213Y-PRPH2 in the inner segment (Fig. 7E). In addition, black arrows in Fig. 7G highlight example areas of colocalization between PRPH2 and calreticulin, indicating that a small but appreciable fraction of the C213Y protein accumulated in the ER. Figure 7H, H′ further illustrate the abnormal localization of PRPH2 in the *C213Y/C213Y* retina, including a pronounced thick perinuclear ring of PRPH2.

As in the WT rods, the *Y141C/C213Y* and *C150S/C213Y* mutant rods only showed visible inner segment PRPH2 in images with intensity levels that saturated outer segments (Fig. 7I vs. Figure 7J and Fig. 7M vs. Figure 7N) and like the WT, these models exhibited very little colocalization between PRPH2 and calreticulin (Fig. 7K, O). However, we observed a perinuclear ring of PRPH2 in both the *Y141C/C213Y* and the *C150S/C213Y* (similar to the *C213Y/C213Y*) suggesting that while most PRPH2 in these models targeted to the OS, some fraction was mistrafficked (Fig. 7L, P).

We next co-labeled compound mutant retinas and homozygotes for PRPH2 and ROM1 (Fig. S8). In WT retinas, PRPH2 and ROM1 colocalized throughout the outer segment and this pattern was consistent in all compound and homozygous retinas except in the *C213Y/C213Y.* In *C213Y/C213Y* retinas, ROM1 was targeted to the outer segment while C213Y-PRPH2 was retained in the inner segment (Fig. S8, right).

**Fig. 8:**
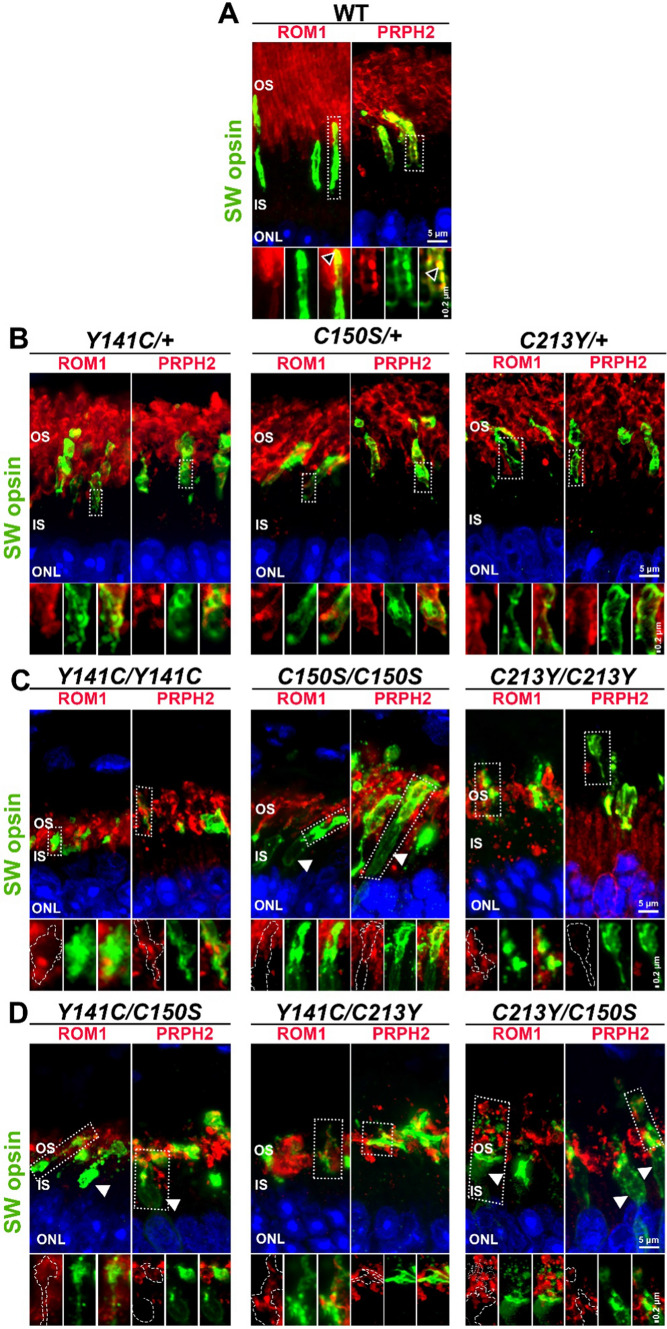
Cone outer segment trafficking of *Prph2* mutants. **A–D** P30 retinal cross sections were co-labeled for SW opsin (green) and either for ROM1 or PRPH2 (red), to evaluate the presence of PRPH2 and ROM1 in cone outer segments. Bottom panels are captures of boxed regions. White dashed lines are traces of cone outers segments overlaid onto signal channel images of PRPH2 and ROM1, to assess the localization of PRPH2 in the cone. White arrowheads point to instances of mislocalized SW opsin. Black arrowheads point to instances of colocalization. Scale bar in low magnification panels is 5 µm. Scale bar in insets is 0.2 µm. *OS* outer segment, *IS* inner segment, *ONL* outer nuclear layer

To understand whether the C213Y-associated trafficking defects also occurred in cones, retinas were co-labeled for short wavelength (SW) opsin and either PRPH2 or ROM1 (Fig. 8). In WT cones (Fig. 8A, bottom panels), ROM1 and PRPH2 were located to the cone outer segments (example areas of colocalization marked by black arrowheads). PRPH2 and ROM1 were also found in cone outer segments in heterozygous retinas, though the outer segments appeared in irregular shapes (Fig. 8B). In the homozygous retinas, cone outer segments were even more dysmorphic than in heterozygous retinas, so traces of the cones were overlaid onto single channel images of PRPH2 and ROM1 (bottom panels, Fig. 8C, D) to better evaluate their presence in these abnormal outer segment structures. In *Y141C/Y141C* retinas, PRPH2 and ROM1 were inside the cone trace and no opsin mislocalized to the inner segment (as previously observed in Fig. 3D). *C150S/C150S* retinas, on the other hand, showed mislocalization of SW opsin to the inner segment, appearing to highlight the cone inner segment (white arrowheads, Fig. 8C and as seen in Fig. 3D). However, ROM1 and PRPH2 were localized in the *C150S/C150S* outer segment and did not mislocalize to the inner segment with SW opsin (Fig. 8C, bottom panels), indicating that while the presence of C150S protein led to improper opsin localization, PRPH2 and ROM1 targeting/trafficking in the cones were not affected. In *C213Y/C213Y* cones, PRPH2 behaved as in rods, and the cone outer segment was devoid of PRPH2 labeling.

Cones in both *Y141C/C150S* and *C213Y/C150S* retinas adopted features of the *C150S/C150S* phenotype (Fig. 8D). White arrowheads point to areas of cone opsin mislocalization as previously observed (Fig. 3E). In *Y141C/C150S,* ROM1 and PRPH2 were seen within the boundary of the cone trace and mislocalized with SW opsin to the inner segment. Cone outer segments in *Y141C/C213Y* retinas were dysmorphic, but PRPH2 and ROM1 were found within the cone traces (Fig. 8D, center of bottom panels). In the *C213Y/C150S* retina, cone outer segments were dispersed and unstructured and SW opsin mislocalized to the inner segment. As a result, it was difficult to evaluate the localization of PRPH2 and ROM1 in these cells, though it was clear that immunolabeling of PRPH2 and ROM1 was present in the outer segment space.

### Homozygous and compound *Prph*2 mutants maintain interactions with Syntaxin 3

**Fig. 9:**
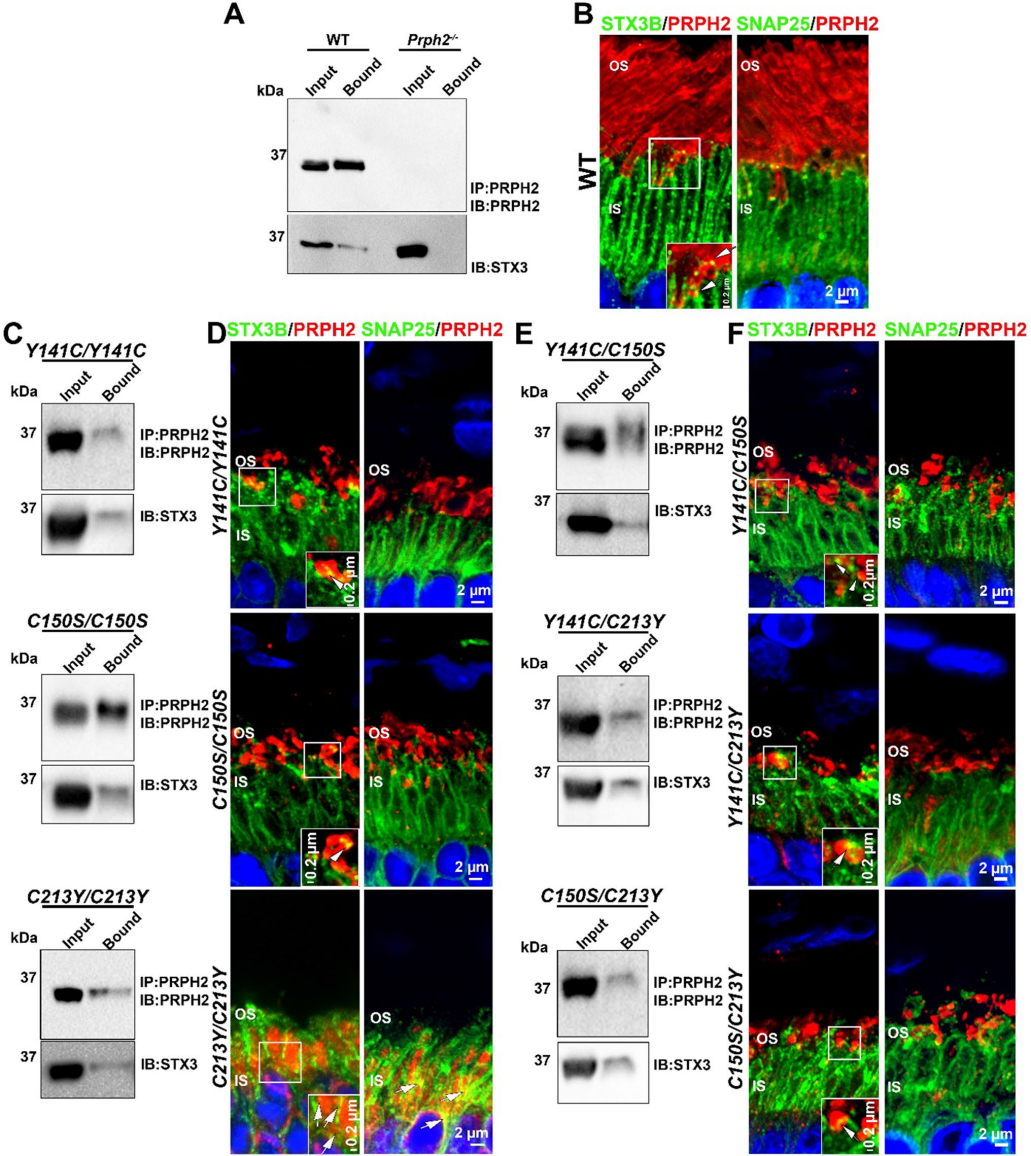
Cysteine mutants maintain interactions with STX3B. **A**, **C**, **E** Immunoprecipitation (IP) was performed on P30 retinal extracts using anti-PRPH2 antibody. Blots with “Input” and “Bound” were probed (IB) with antibodies against PRPH2 and STX3, highlighting the ability of PRPH2 to interact with STX3 in all lines. **B**, **D**, **F** P30 retinal cross-sections were labeled for PRPH2 (red) and either STX3B or SNAP25 (green). Boxed areas are shown larger in insets, arrows highlight regions of colocalization. Images labeled with STX3B and SNAP-25 were captured at ×63 with ×2.5 zoom. Scale bar is 2 µm in STX3B and SNAP-25 labeled images and 0.2 µm in insets. *OS* outer segment, *IS* inner segment, *ONL* outer nuclear layer

We have previously demonstrated that the SNARE proteins, Syntaxin 3B (STX3B) and synaptosome-associated protein 25 (SNAP25), interact with PRPH2 and are important for transporting it to the plasma membrane at the ciliary base during outer segment targeting [67, 68]. So, to follow our evaluations of trafficking behavior in the mutant retinas, we performed immunoprecipitation with an anti-PRPH2 antibody, with *Prph2*^*−/−*^ included as a negative control (Fig. 9A), to determine whether mutant proteins with trafficking defects retain the ability to interact with STX3. In the WT, homozygous, and compound mutant retinas PRPH2 bound STX3 (Fig. 9A, C, E). Despite the variability in the yield of the immunoprecipitation, it is clear that each mutant maintains some capacity to bind to STX3.

Since mutant PRPH2 in the *C213Y/C213Y* retina did not traffic to the outer segment in either rods or cones and accumulated in the inner segment, it was surprising to find out that it retained its interaction with STX3B. To further explore potential trafficking defects in our homozygous and compound mutant retinas, retinas were immunofluorescently labeled for PRPH2, STX3B and SNAP25 (Fig. 9B, D, F). In the WT retina, both STX3B and SNAP25 localized along the inner segment plasma membrane (Fig. 9B). STX3B was also observed at the apical edge of the inner segment where trafficking vesicles fuse with the plasma membrane (Fig. 9B, inset). Although outer segments were much shorter in the *C150S/C150S*, *Y141C/Y141C*, and *Y141C/C150S*, this labeling pattern was maintained (Fig. 9D, F with insets showing small pools of colocalization between PRPH2/STX3B). In *C213Y/C213Y* retinas we observed small areas of colocalization primarily along the inner segment plasma membrane (Fig. 9D, inset, bottom-most panel), with the strongest colocalization signal appearing between SNAP25 and PRPH2 (yellow regions indicated with white arrows, Fig. 9D, lowest image, right-most), where we also observe yellow signal perinuclearly. The immunoprecipitation and colocalization between PRPH2, SNAP25, and STX3B suggests that C213Y-PRPH2 may be packaged into post-ER/Golgi vesicles that can associate with the T-SNARE complex on the inner segment membrane, but proper fusion and downstream mechanisms are affected preventing further processing along the outer segment targeting pathway. This dramatic defect in C213Y outer segment trafficking was largely corrected in the *Y141C/C213Y* and *C150S/C213Y* compound mutant retinas where there were colocalization of STX3B with PRPH2 at the apical inner segment membrane (white arrow in the insets of Fig. 9F, middle and lower, left images) and localization of PRPH2 at the outer segment (Fig. 9F).

### Compound mutant retinas demonstrate dysmorphic outer segment structure

**Fig. 10:**
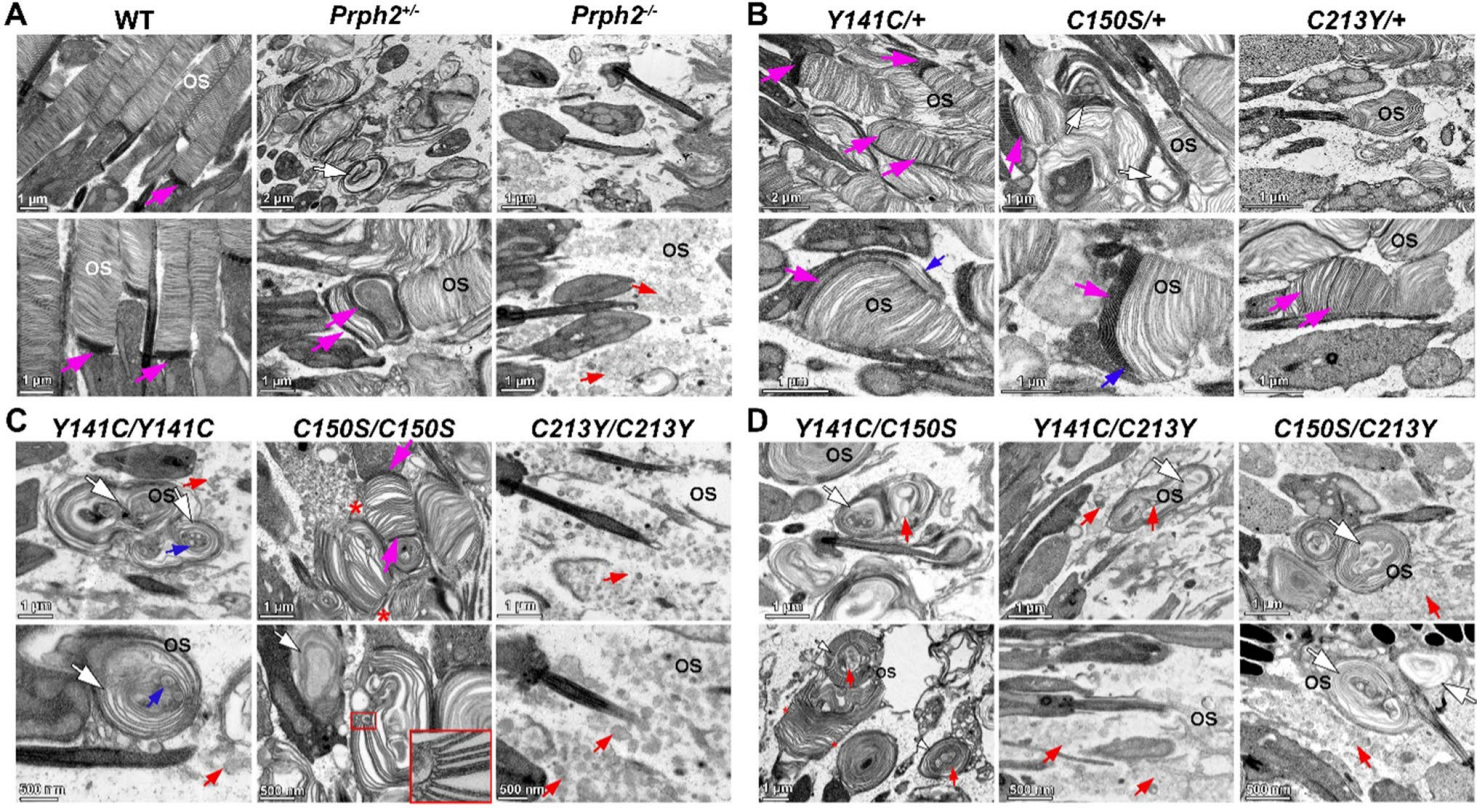
Compound mutants generate highly dysmorphic outer segment structures. **A–D** Transmission electron microscopy (TEM) on tannic acid/uranyl acetate stained retinal sections was used to evaluate outer segment ultrastructure at P16. Arrows point to important abnormal features of degenerated retina: magenta arrows: open/nascent discs (tannic acid stained), blue arrows: disc overgrowth, white arrows: whorls, red arrows: ectosomes/entrapped vesicles, red asterisks: rims. Scale bars are labeled in each image.

To understand how changes in oligomer distribution and protein trafficking influenced photoreceptor outer segment ultrastructure, transmission electron microscopy was performed on retinas stained with tannic acid and uranyl acetate at P16 (Fig. 10) and P30 (Fig. S9). Tannic acid is a contrasting agent that can be used to stain the nascent open discs generally found at the base of the outer segment in WT retina [69] (magenta arrows Fig. 10A, left panels).

In the haploinsufficient *Prph2*^+/−^, outer segments were present as whorls (white arrow Fig. 10A, middle), discs were disorganized, misaligned, there were irregular large gaps between disc strata, and the morphology of the open discs at the base of the outer segments was highly abnormal (magenta arrows) as previously described [41, 43]. In the heterozygous mutant retinas, OS structure was also abnormal, either from dosage effects or due to toxic gain-of-function and dominant-negative mutations [39, 50] or a combination. *Y141C*/+ retinas had essentially normal levels of protein (per cell), but many outer segments were irregular, misaligned, (Fig. 10B, left white arrow), and exhibited a greater number of open discs than normal (magenta arrow), and overgrowth of newly formed discs (bottom panel, blue arrow) [38]. There also appeared to be a banding pattern of open discs in one instance, suggesting irregularities in the disc closure process. In *C150S*/+ retinas, there are whorled structures (white arrows), misaligned discs, and a basal accumulation of open discs. In fact, despite having a different oligomeric composition to *Y141C*/+, some aspects of the ultrastructure of *C150S*/+ outer segments were very similar to the *Y141C*/+ mutant retina*.* In the bottom panel of Fig. 10B (left-most and middle) we observed outer segments of both *Y141C*/+ and *C150S*/+ exhibiting basal accumulation of open disc and disc-overgrowth. In the *C213Y*/+ retina (Fig. 10B, right), we observed smaller, more dysmorphic outer segment structures, in which discs were misaligned and poorly formed. Tannic acid staining revealed that some open discs (magenta arrows) were occasionally interspersed among closed discs, indicating abnormalities in the disc enclosure process, analogous to outer segments in *Y141C*/+ retina.

Homozygote retinas, exhibited profoundly dysmorphic outer segment structures (Fig. 10C). *Y141C/Y141C* outer segments were entirely overgrown into whorls (white arrows). As in the *Prph2*^*−/−*^ (Fig. 10A, right and [70]) an appreciable amount of extracellular vesicles/ectosomes were present in the *Y141C/Y141C* mutant retina (Fig. 10C, red arrow) surrounding the outer segment whorls, occasionally vesicles were entrapped within the whorls (blue arrows). Photoreceptors of the *C150S/C150S* retinas retained some disc architecture, unlike *Y141C/Y141C* retinas. Rim formation was present (red stars, and inset), and in some cases, normal disc stacking was observed. There was even enough structure to discern a banding pattern of open and closed discs (albeit abnormal, magenta arrows). However, discs were mostly misaligned, abnormally sized, and in a whorl-like configuration (white arrow). *C213Y/C213Y* retinas were indistinguishable from the *Prph2*^*−/−*^ with no sign of outer segment formation and the subretinal space filled with ectosomes (Fig. 10C, right, red arrows).

The outer segments of compound mutant animals, like their homozygous counterparts, had significantly compromised ultrastructure (Fig. 10D and Fig. S9). The *Y141C/C150S* produced predominantly whorled structures (white arrows). On occasion, *Y141C/C150S* outer segments exhibited nicely formed disc rims and stacked, properly aligned discs (red stars, Fig. 10D). Overall, *Y141C/C150S* outer segments looked slightly less dysmorphic than *Y141C/Y141C* outer segments. There were fewer ectosomes than in the other compound mutant retinas, suggesting that a sufficient level of PRPH2 was produced and trafficked to the outer segment to support initial outer segment formation.

In the *Y141C/C213Y* retina (Fig. 10D and S9) outer segments were more dysmorphic than in the *Y141C/Y141C*. The few outer segments that were formed exhibited small-sized whorls (white arrows) often accompanied by entrapped vesicles. Many connecting cilia had no outer segment membranes at all and significant ectosome accumulation was observed in the subretinal space (red arrows). Similarly, there was an appreciable accumulation of ectosomes in *C150S/C213Y* retinas (Fig. 10D and S9). However, whorled structures were also quite frequent in these mice (white arrows). Ectosomes were not observed in heterozygous retinas, consistent with the fact that ectosomes result from a significant loss of PRPH2 and its ability to retain membranous material at the primary cilium (e.g., in the *Prph2*^*−/−*^ or homozygous and compound retinas).

## Discussion

**Fig. 11:**
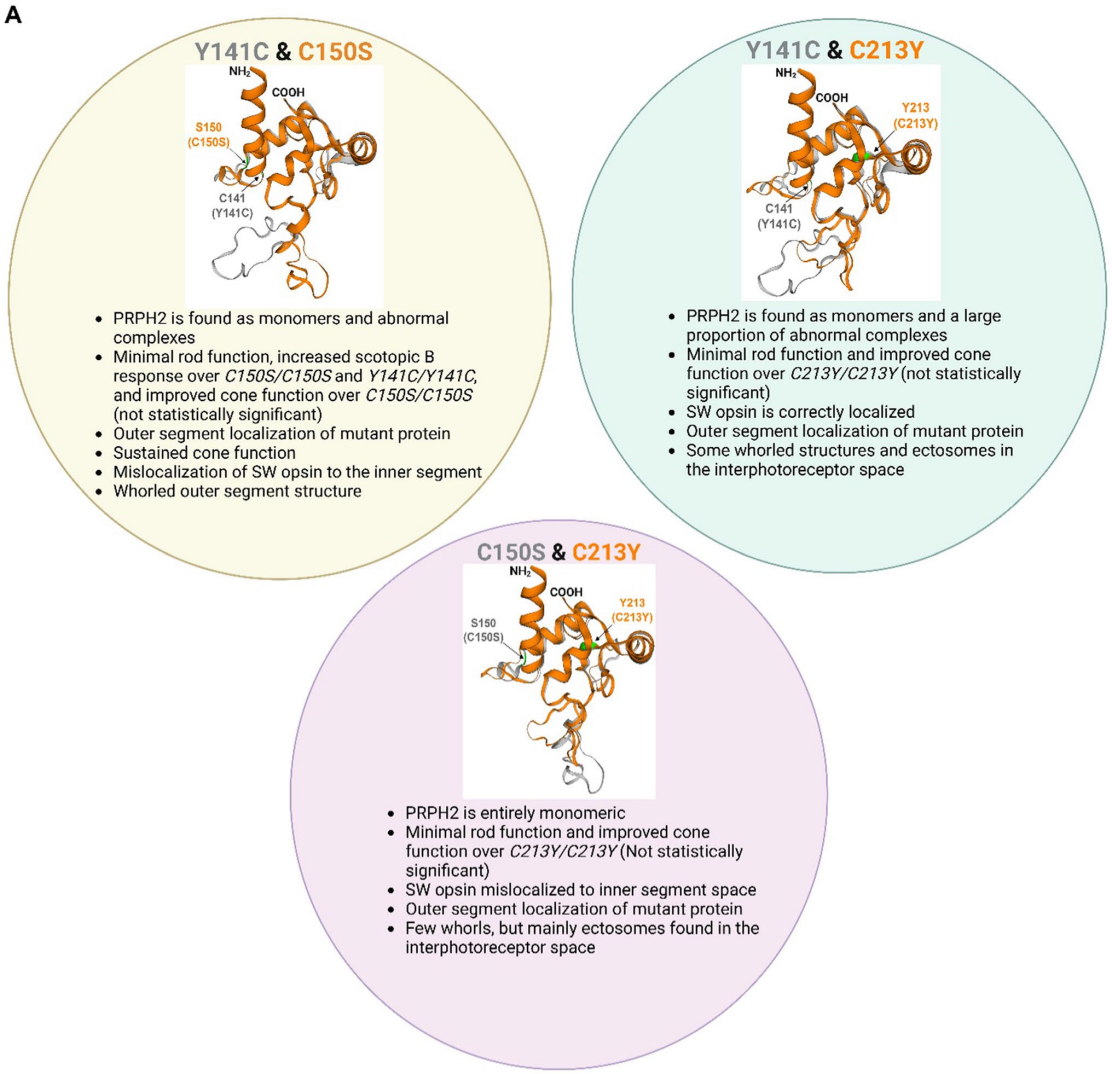

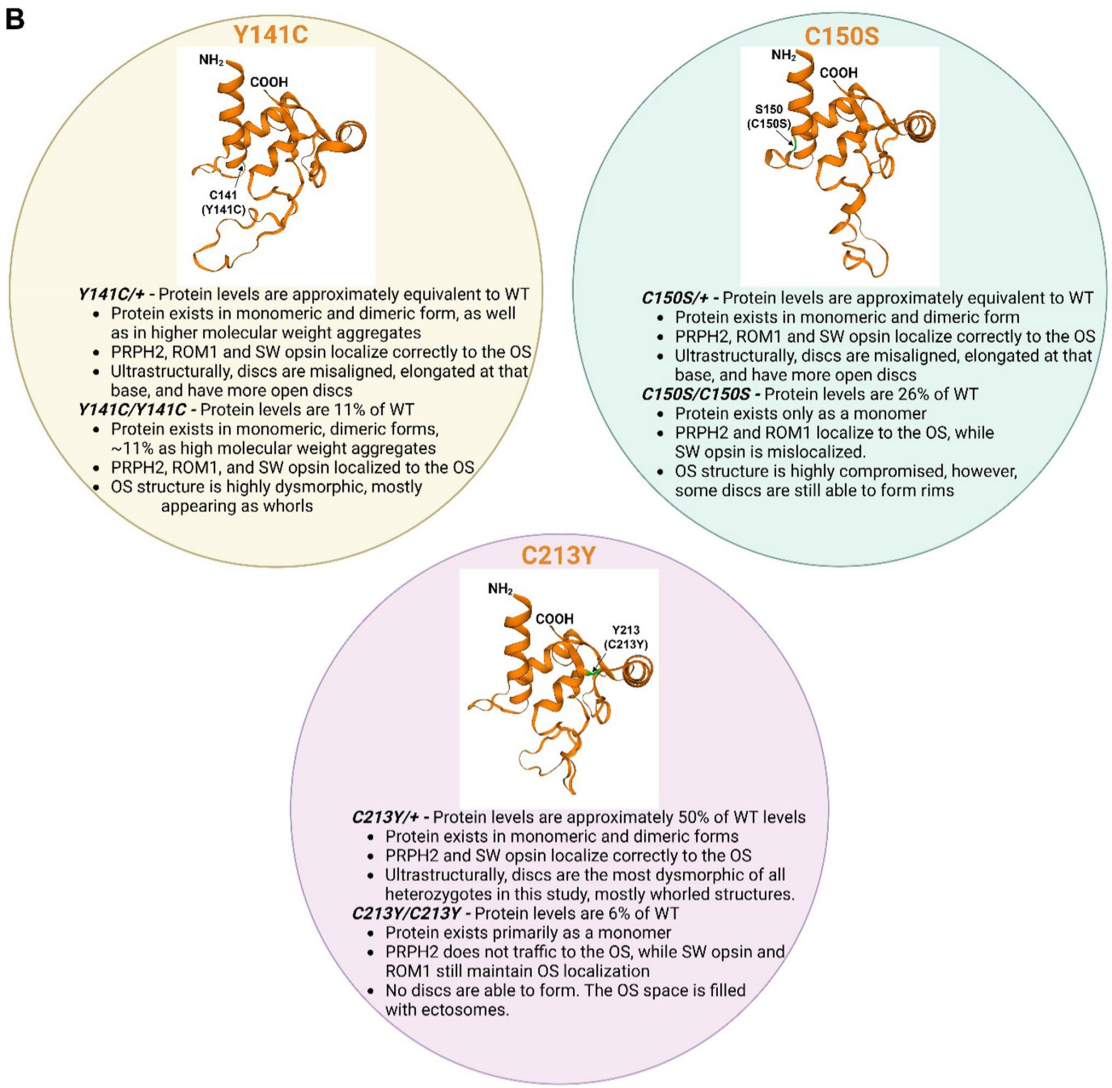
Structural comparison of mutant D2 loop and summary of observations. **A, B **The same prediction tool as used in Fig. S2 was adopted to compared the D2 loop structure in different Prph2 mutants. Bullet points describe critical properties of each mutation. **B** The colors in the title of each mutant comparison correspond to the colors used in the overlay. Created with BioRender.com

In this study, we analyzed the functional, structural, and biochemical consequences of PRPH2 D2 loop mutations, alone and in combination with each other in order to refine our framework for understanding mechanisms that cause retinal disease. Figure 11 and Table 1 summarize our findings and highlight the predicted conformational differences in D2 loop structure between the mutants. Divergent regions between mutants occur primarily in the large loop between H3 and H4 in Y141C and C213Y, and close to H4 near the C-terminal region of D2 loop (Y141C and C150S) (Fig. 11A). These predicted structural changes likely underlie the significant alterations to the biochemical properties of the PRPH2 protein seen in mutant retinas, in particular the ability of PRPH2 and ROM1 to form non-covalent interactions is still present in mutants, but the larger complex assembly is disrupted, suggesting the novel finding that proper structure of the hypervariable region between H3 and H4 in D2 loop is essential for assembly of correct PRPH2 complexes. These biochemical defects ultimately lead, through their effects on outer segment structure, to reduced visual function. In principle, reduced visual function in *PRPH2*-associated diseases may come from reduced phototransduction. However, our data suggest that this impairment occurs both because of cellular degeneration and defects in the outer segment structure that reduce the outer segment volume (thus reducing the quantity of phototransduction components) and impaired outer segment organization (thus reducing the efficiency of phototransduction). These data shed light on how different PRPH2 mutations can affect these parameters differentially, thereby highlighting several novel or little discussed disease mechanisms. Major findings for each genotype are listed in Table 1.

**Table 1:** Summary of compound mutant properties

Genotype	Amount of PRPH2 (% WT)	Oligomerization	PRPH2 OS localization	OS ultrastructure	P30 rod function (% WT)	P30 cone function (% WT)	Cone opsin localization	SNARE association
WT	100%	Monomer/dimer	Yes	Normal discs	100	100%	Proper localization	Yes
*Y141C/Y141C*	11%	Monomer/dimer/aggregate	Yes	Whorls/ectosomes	8%	45%	Proper localization	Yes
*C150S/C150S*	26%	Monomer	Yes	Whorls/some disc stacking	20%	33%	Partially mislocalized	Yes
*C213Y/C213Y*	6%	Monomer/dimer	No	Ectosomes	7%	24%	Proper localization	Yes
*Y141C/C150S*	24%	Monomer/aggregate very little dimer	Yes	Whorls/ectosomes	17%	39%	Partially mislocalized	Yes
*Y141C/C213Y*	14%	Monomer/dimer/aggregate	Yes	Ectosomes/whorls/	7%	45%	Properly localized	Yes
*C150S/C213Y*	8%	Monomer	Yes	Ectosomes/whorls	8%	37%	Partially mislocalized	Yes

Table provides comprehensive summary of compound mutant properties in comparison to WT and corresponding homozygotes. Properties include PRPH2 protein level (as % of WT), oligomerization, outer segment localization, ultrastructural phenotype, rod and cone function (ERG amplitudes as % of WT), localization of cone opsin, and SNARE association

One key finding from this study is that the pathology of mutant *PRPH2*-associated rod disease is not entirely due to haploinsufficiency (i.e., reductions in PRPH2 levels). Haploinsufficiency preferentially targets rods [43], but our current data demonstrate that severe defects in rod function are also apparent in models that do not exhibit significant haploinsufficiency. It has been previously shown that ~ 80% of WT PRPH2 levels are needed for full rod function [71], and here we found that models without significant reduction in cellular PRPH2 level such as the *Y141C*/+ and *C150S*/+ exhibit severe defects in rod function, clearly indicating that gain-of-function defects or dominant-negative effects/haploinsufficiency adversely impact rods. This defect in rod function is likely tied to the reduced ROM1 levels that occur even in the mutant PRPH2 heterozygous retinas that do not exhibit large decreases in PRPH2 levels. These rod phenotypes are also consistent with what is reported in patients; for example, Y141C patients exhibit rod defects [23, 72, 73].

A closely related finding highlighted by this work is that most PRPH2 mutations cause both rod and cone defects. The initial paradigm that some mutations target rods while others target cones originated from data gained from various transgenic and knockout models [31, 42, 43, 46, 47, 74]. Though informative, these studies suffered from challenges associated with the use of transgenics such as non-native gene regulation and variable levels of transgene expression. Indeed, by using our knockin models we find that disease-causing mutations affect both rods and cones. This clearly models what occurs in patients, and many patients, even those with cone-related macular or pattern dystrophy, also exhibit defects in rods [22, 75]. Overall, we found that PRPH2 mutations are much more toxic to rods than cones (measured in terms of rod vs. cone function and cell loss). Even for mutations like C150S, in which cones were affected more than in other mutant retinas, the defects in rods were most severe. This ability of cones to function better than rods in the presence of mutant PRPH2 is supported by our prior observation that in the *Prph2*^*−/−*^*/Nrl*^*−/−*^ retina, cones still exhibit phototransduction and form balloon like membrane structures even though the absence of PRPH2 means that no flattened lamellae are formed [45]. It could simply be that the different morphology of the cone outer segment where lamellae are rimmed at one end, and open to the extracellular space at the other [76] makes cones less reliant on PRPH2 [77]. This is supported by findings that cones are less affected by PRPH2 haploinsufficiency than rods [43, 78]. From a clinical context, these results suggest that correcting cone defects, by knocking down mutant alleles to eliminate gain-of-function/dominant-negative effects, may be easier than correcting rod defects since rods suffer from both gain-of-function/dominant-negative effects and haploinsufficiency.

The next disease mechanism to highlight arises from our work using the *Y141C/C213Y* compound mutant retina. While this mutant retina (like all the compounds and homozygotes) had very poor photoreceptor structure and function, as well as significant degeneration, subtle differences between the *Y141C/C213Y* and *C213Y/C213Y* genotypes shed light on the role of inner segment accumulation of PRPH2 in promoting photoreceptor degeneration. We had previously hypothesized that the accumulation of C213Y protein in the inner segments contributed to the toxic phenotypes in the *C213Y/C213Y* retina, but it was difficult to verify, because the *C213Y*/+ had radical improvements in many cellular outcomes compared to the *C213Y/C213Y* (i.e. protein levels, complex assembly, and most notably outer segment structure). Here, we found that at early time points, retinal degeneration is less severe in the *Y141C/C213Y* retina than in the *C213Y/C213Y* retina and that scotopic and photopic b-waves are slightly higher in the *Y141C/C213Y* vs. *C213Y/C213Y*. This phenotypic difference cannot be attributed to improved PRPH2 complex assembly; the *Y141C/C213Y* exhibits an increase in the presence of abnormal high molecular weight aggregates that are toxic in Y141C retinas. This could be attributed to the finding that outer segment structure is slightly improved in the *Y141C/C213Y vs. C213Y/C213Y*, but the few outer segments structures we observed were very small and intermittent. The most dramatic phenotype in which *Y141C/C213Y* exhibited improvement compared to *C213Y/C213Y* was in the reduced accumulation of PRPH2 in the inner segment. It is not possible to distinguish whether this arises due to increased trafficking of C213Y to the outer segment with Y141C or simply because there is less C213Y protein overall, but these data clearly suggest that preventing PRPH2 from accumulating in the inner segment promotes photoreceptor survival. Support for the idea that inner segment accumulation of mutant PRPH2 is toxic to photoreceptors comes from our finding that the *C213Y/C213Y* retina degenerates even faster than the *Prph2*^*−/−*^.

Despite the small degree of benefit seen in the *Y141C/C213Y* vs. *C213Y/C213Y* genotypes, both *Y141C* and *C213Y* mutations are highly deleterious. We were initially interested in these mutations because both affected D2 loop cysteines, however *in vitro* expression of *Prph2*^*Y141S*^ and *Prph2*^*Y141F*^ mutants resulted in high molecular weight aggregates similar to Y141C [79], suggesting that introduction of an extra cysteine is not the central cause of abnormalities in oligomerization in *Y141C* animals. Additional support for this idea comes from the observation that *Prph2*^*C214S*^ [63] and *Prph2*^*K153del*^ [80] also form large aggregates. These high molecular weight complexes are covalently linked based on their absence under reducing conditions, so it is possible that the destabilizing effect of the *Y141C* mutation highlighted in Fig. 1 induces a detrimental change in folding freeing a cysteine at another position in the D2 loop to form aberrant disulfide bonds. *C213Y*, on the other hand, causes an even greater degree of instability; it has the largest negative ΔΔG of all mutations in the study, and caused the most severe phenotypes-significantly reduced expression levels, no disc elaboration, and completely impaired PRPH2 outer segment trafficking. C213 is part of a highly conserved tetraspanin motif (PxxCC) in the D2 loop [24, 59–62], and mutations identified in this motif such as C214S and P210L cause severe degeneration [63, 64] and inner segment mislocalization of PRPH2 [81]. The role of this conserved domain is unknown, but it is hypothesized that mutations in this region affect the distal part of the D2 loop [82]. The precise role of the distal part of the D2 loop is not known, but it is part of the hyperconserved region among tetraspanin superfamily members suggesting it is important for overall protein function.

The *Y141C/C150S* retinas harbor more protein than would be predicted based on levels in the *Y141C/Y141C* and *C150S/C150S*. However, it is not clear whether this is because C150S PRPH2 can stabilize Y141C protein or because the *Y141C/C150S* mutant retina has improved outer segment structure and thus increased volume for PRPH2 accumulation. Of the mutant proteins utilized in this study, C150S was expressed at the highest level. Though C150S lacks the ability to form intermolecular covalent disulfide bonds, it forms normal PRPH2 tetramers [40]. The *C150S/C150S* homozygous retinas exhibited outer segment structures that were better than the other two homozygous mutant retinas, confirming the idea that tetramers are capable of initiating disc morphogenesis, but large covalent oligomers are needed to fully form proper outer segments [40]. However, Y141C protein can also form tetramers, and while some outer segment structures are seen in *Y141C/Y141C* rods, they are smaller and more dysmorphic than those in *C150S/C150S* rods. This is likely due to the abnormal high molecular weight aggregates in the Y141C which may be toxic or simply useless for providing outer segment support.

Many of the observations discussed here are predicated on the assumption that mutant proteins maintain some capacity to interact with each other, make homomeric interactions, or interact with the WT protein. Further studies are ongoing to provide direct evidence to this effect, however, experimental challenges such as the generation of antibodies capable of distinguishing between the WT and mutant PRPH2 make this kind of investigation quite difficult in vivo. Nevertheless, prior studies have provided preliminary/indirect evidence that mutant-WT or mutant-mutant interactions do occur. In vitro studies using epitope tags have shown that C150S and Y141C-PRPH2 do make associations with themselves, each other, and with the WT PRPH2 [40]. Furthermore, homomeric PRPH2 interactions have also been validated in *Y141C/Y141C* and *C213Y/C213Y* retinas by velocity sedimentation experiments. Specifically, PRPH2 tetramers and higher order complexes were detected in *Prph2*^*Y141C/Y141C*^*/Rom1*^*−/−*^ retinas and in *Prph2*^*C213Y/C213Y*^ retinas [50, 79] The C213Y protein does not bind ROM1, so any PRPH2 complexes present in *C213Y/C213Y* retinas reflect homomeric interactions. In addition, based our observations here, there is some evidence to suggest that there are interactions between Y141C and C213Y-PRPH2 and between C150S and C213Y-PRPH2, since we find that that PRPH2 is no longer retained in the inner segment in *Y141C/C213Y* and *C150S/C213Y* retinas (compared to *C213Y/C213Y*), suggesting that C213Y is trafficked out of the inner segment due to interactions with C150S and Y141C. However, it is also possible, that the small amount of C213Y-PRPH2 protein is simply degraded in the compound mutant retinas rather than being properly trafficked. Future studies are needed to fully understand this picture.

Ultimately, our study comparing three different D2 loop cysteine mutations expressed alone and in combinations highlights two critical disease mechanisms relevant to the development of *PRPH2*-associated inherited retinal diseases. We provide biochemical and cellular data showing that rod-dominant diseases are not only associated with haploinsufficiency, but also that rods can be severely affected by gain-of-function/dominant-negative mutations like Y141C and C150S. Our data also demonstrate that cones are more resilient to PRPH2 mutations than rods. Whereas many PRPH2 mutations cause macular dystrophy, our updated understanding of disease mechanisms from this and previous studies suggests that macular disease likely arises after abnormalities in outer segment structure. This consequently leads to defects in adjacent tissues rather than to cone-specific defects in photoreceptors [28, 83]. Overall, these findings significantly refine our understanding of *PRPH2*-associated disease mechanisms and highlight how sensitive rod photoreceptors are to alterations in the structure and quantity of PRPH2.

## Materials and methods

### Mouse lines

All animals and experimental procedures were approved by the University of Houston Animal Care and Use Committee (IACUC), and adhered to the guidelines provided by the Association for Research in Vision and Ophthalmology and the National Association for Research in Vision and Ophthalmology Resolution on the Use of Animals in Research. Knockin *Prph2* mutants, *Y141C, C213Y*, and *C150S* were generated by Ingenious Targeting Laboratory, Inc. (Ronokoma, New York, NY, USA). Genetic strategy and characterization were previously described [39, 40, 50]. Animals were maintained on an in-house WT background. The WT line was generated by crossing C57BL/6 with FVB animals, and backcrossing several generations to C57BL/6 to generate animals negative for the *rd1* and *rd8* alleles*,* and carrying the *RPE65*^*Leu*^ variant on a C57BL/6 background. Animals were kept under standard lighting conditions, 12 h light (~ 30 lx) and 12 h dark. Animals euthanized for tissue collection were asphyxiated by CO_2_ followed by cervical dislocation. No differences were noted with the presented phenotypes between the sexes.

### Protein extraction, immunoblotting and protein quantification

Retinas were collected between postnatal day (P) P21 and P30 and flash frozen in liquid nitrogen and stored at ultralow temperature until use. Frozen retinas were lysed in cold buffer consisting of 1× PBS pH 7.0 containing 1% triton X-100, 5 mM EDTA, 5 mg/ml n-ethylmaleimide, and protease inhibitor (Complete™ Protease Inhibitor Cocktail, Roche, Indianapolis, IN, USA). One hundred µl aliquot of lysis buffer per retina was used for WT and heterozygous samples, while 50 µl of lysis buffer per retina was used for compound mutants and homozygous samples. Samples were briefly homogenized with a hand-held homogenizer and sonicated, then incubated at 4 °C for 2.5 h. Homogenized samples were centrifuged at 25,000×g for 15 min (5424R Eppendorf, Enfield, CT, USA) and supernatants were collected. Bradford assay was performed according to manufacturer instructions to determine protein concentration (Bradford reagent, Bio-Rad, Hercules, CA). Between 15 and 20 µg aliquots of protein were loaded for WT and heterozygous samples and 35–50 µg of protein was used for homozygous and compound mutants samples. β-mercaptoethanol (BME) (M6250, Sigma Aldrich, Burlington, MA) was excluded from sample preparation for non-reducing conditions. All samples were loaded onto 11% polyacrylamide gels. Immunoblotting, imaging, and densiometric analyses on non-saturated blots were performed as previously described [50]. Immunoblots were probed with antibodies listed in Table 2.

**Table 2:** List of antibodies used in this study

Antigen	Species	Clone	Application/concentration	Source
Primary antibodies				
PRPH2	Rabbit	RDS-CT	1:1000 (WB), 1:500 (IF)	In house [39] RRID: AB_2833006
PRPH2	Mouse	2B7	1:1000 (WB). 1:500 (IF)	In house [77] Available at Millipore Cat# MABN2395
ROM1	Mouse	2H5	1:500 (WB and IF)	Invitrogen Cat# L21409 RRID: AB_2315178 [83]
Acetylated alpha tubulin	Mouse	6-11B-1	1:250 (IF)	Sigma-Aldrich T6793 RRID:AB_477585
SW opsin	Rabbit		1:500 (WB)	Millipore Sigma AB5407 RRID:AB_177457
Syntaxin 3	Rabbit		1:250 (IF), 1:500 (WB)	Novus Biologicals NBP1-86984 RRID:AB_11055708
Syntaxin 3B	Mouse	12E5	1:500 (IF)	In house [67]
SNAP25	Rabbit		1:500 (IF)	Proteintech 14903-1-AP RRID:AB_2192051
Calreticulin	Chicken		1:500 (IF)	Invitrogen Cat# PA1-902A RRID: AB_2069607
Β-actin	Mouse		1:15,000 (WB)	Millipore Sigma Cat# A3854 RRID:AB_262011
Secondary antibodies				
Anti-mouse-HRP	Goat		1:25,000 (WB)	Sigma Aldrich, Cat# AP130P RRID:AB_91266
Anti-rabbit-HRP	Goat		1:25,000 (WB)	Sigma Aldrich, Cat# AP187P RRID:AB_92625
Anti-mouse AF555	Donkey		1:500 (IF)	Thermo Fisher Cat# A32773 RRID:AB_2762848
Anti-mouse AF488	Donkey		1:500 (IF)	Thermo Fisher Cat# A-21202 RRID:AB_141607
Anti-rabbit AF647	Goat		1:500 (IF)	Thermo Fisher Cat# A32795 RRID:AB_2762835
Anti-rabbit AF555	Goat		1:500 (IF)	Thermo Fisher Cat# A32732 RRID:AB_2633281
Anti-rabbit AF488	Goat		1:500 (IF)	Thermo Fisher Cat# A-11008 RRID:AB_143165
Anti-chicken AF488	Donkey		1:500 (IF)	Thermo Fisher Cat# A78948 RRID:AB_2921070

### Immunoprecipitation

Flash frozen tissue was homogenized in HEPES buffer with CHAPS (20 mM HEPES, 150 mM NaCl, 5 mM CHAPS, and protease inhibitor, pH 7.5). Samples were sonicated and left to solubilize overnight at 4 °C. The following day samples were spun down at 25,000×g for 15 min. and protein concentration was determined by Bradford assay. To ensure effective pulldown, 250 µg of protein was used for WT samples, and 450–500 µg of protein were used for homozygous and compound mutant samples. Immunoprecipitation was performed according to procedures described in [67]. Protein G beads were acquired from ThermoFisher (15920010, ThermoFisher Scientific, Waltham, MA, USA). PRPH2 2B7 antibody (in house [37, 83]) was used for immunoprecipitation, while anti-Syntaxin 3 (Novus Biologicals, Littleton, CO), and PRPH2-CT (in house [46]) were used for immunoblotting (see table for dilutions). One third of total eluent was loaded for “bound” samples while 10–20% of total protein used for the immunoprecipitation was loaded as “input”.

### Tissue fixation, and histology

Following euthanasia, the cornea was marked to indicate the superior orientation. The globe was carefully removed from the ocular cavity and fixed in a modified Davidson fixative (32% ethanol, 11% acetic acid, 2% formaldehyde) for immunofluorescence and morphometry. Once removed from the fixative, eyes were rinsed in 1× PBS three times and then placed in a STP 120 Spin Tissue Processor (Thermo Scientific, Waltham, MA, USA). Tissue processor was programmed according the dehydration protocol described in [84]. After dehydration and paraffinization, eyes were embedded in plastic molds (Tissue Tek Sakura, Finetek, Torrance, CA, USA) and left to harden. Paraffin sections (10 µm) were cut along the superior-inferior axis. Sections containing the optic nerve were used for morphometry and histological analyses, remaining sections were used for immunofluorescent labeling.

Deparaffinized retinal cross sections containing the optic nerve were stained with hematoxylin (MHS16, Sigma, Burlington, MA, USA) and eosin (HT110116, Sigma, Burlington, MA, USA). Images were captured at 200 µm increments along the inferior-superior axis using a Zeiss Axioskop 50 (Carl Zeiss, Inc. White Plains, NY, USA). Cells of the outer nuclear layer were counted within a 100 µm window every 200 µm. Three independent retinas were counted per genotype and per time point.

### Immunofluorescence

Slides were processed as described above, and were then deparaffinized. Subsequently, slides were boiled in Tris–EDTA buffer pH 9.0 (10 mM Tris, 1 mM EDTA) with 0.05% Tween for 30 min. The slides were left to cool for 20 min then treated with 1% sodium borohydride for 5 min. Slides were washed with 3 changes of deionized water and 3 changes of 1× PBS, then blocked with a buffer containing (5% bovine serum albumin, 10% donkey serum, 5% FBS, 0.1% fish gelatin, and 0.5% TritonX-100 in 1× PBS). Samples were blocked overnight, and the following day primary antibodies were applied and incubated overnight at 4 °C. See Table 2 for full list of antibodies and concentrations. After washing in 1X PBS, appropriate secondary antibodies and DAPI (D1306, ThermoFisher Scientific, Waltham, MA, USA) were applied for 3 h at room temperature. After additional washes in 1× PBS slides were mounted with ProLong Gold anti-fade mounting media (P36931, Thermo Fisher, Waltham, MA, USA). Images were captured using an LSM 800 Zeiss confocal/Airyscan microscope with a 63× oil objective (Carl Zeiss, Inc White Plains, NY, USA). Airyscan is an advanced confocal microscopy with a detector allowing for pinhole-plane images, increasing the precision and resolution at higher magnifications when compared to standard/traditional confocal detection methods [85, 86]. All images presented here are collapsed confocal stacks.

### Electroretinography

Electroretinography was performed according the methods described in [43, 87]. Briefly, following overnight dark adaptation, the pupil was dilated with 2% cyclopentolate (Akorn, Lake Forest, IL USA) for ten minutes and then mouse was anesthetized with intramuscular injection of 85 mg/kg ketamine and 14 mg/kg xylazine (Henry Schein Animal Health, Dublin, OH). The mouse was placed on a warmer set to 38 °C to ensure stable body temperature. Once fully sedated, the mouse was carefully positioned on stage also equipped with a warmer. 2.5% hypermellose ophthalmic demulcent solution (Akorn, Lake Forest, IL, USA) was placed on the cornea followed by the placement of platinum electrodes. The mouse was carefully positioned in the center of the Ganzfeld and exposed to a full-field stimulus of 157 cd·s/m^2^ in scotopic conditions. For photopic recordings, mouse was exposed to a background light of 29.03 cd/m^2^ intensity for five minutes to bleach the retina followed by exposure to 25 white light flashes at 77 cd s/m^2^ intensity.

### Electron microscopy

Eyes were fixed and processed for TEM as previously described [69]. Eyes were collected from transcardially perfused animals. Briefly, animals were anesthetized as described above and perfusion was performed with 2% PFA, 2% glutaraldehyde, and 0.05% calcium chloride in 50 mM MOPS buffer, pH 7.4. After exsanguination, eyes were removed from the ocular cavity and fixed for 2 h in the fixative described above. Eyes were then dissected to remove lens and cornea and then embedded in 2.5% low-melt agarose, and cut into 200 mm thick section with VT1200S Leica Vibratome (Leica Microsystems, Wetzlar, Germany). Agarose sections were then stained with 1% tannic acid (Electron Microscopy Science, Hatfield, PA, USA), 1% uranyl acetate (Electron Microscopy Science, Hatfield, PA, USA), followed by a dehydration step in gradually increasing concentrations of ethanol and then subsequently infiltrated with Spurr’s resin (Electron Microscopy Science, Hatfield, PA, USA). After processing, 70 nm sections were cut, placed on copper grids and counter stained with 2% uranyl acetate and 3.5% lead citrate. Samples were imaged with a JEM-1400 electron microscope at 60 keV with a digital camera (Orius Gatan). Images were viewed and analyzed in ImageJ and Adobe Photoshop (Adobe, San Jose, CA, USA).

### Protein modeling

Using the cryo-EM model of the human PRPH2-ROM1 heterodimer, the PDB file (PDB:7ZW1) was input into PyMOL (Schrödinger, Inc., New York, NY, USA) to visualize the positioning of residues Y141, S150, and Y213. Images of residue-specific regions were generated. The sequence was modified to address each mutation and visualize the effect on interactions with surrounding amino acids. Using the same PDB, the DDGun3D server (https://folding.biofold.org/ddgun/method.html) [53, 54] and PDBePISA (https://www.ebi.ac.uk/pdbe/pisa/) [52] were used to determine the change in Gibb’s free energy of unfolding (ΔΔG) as a consequence of different mutations and the accessible surface area of these amino acid, respectively.

The amino acid sequence of the mouse PRPH2 D2 loop (residues 125–251) was input into the SWISS-MODEL protein homology-modelling server [88, 89]. The web-based tool is automated and provides a list of possible templates after input. The template used was the crystalized structure of the large extracellular loop of Tetraspanin-15 (LEL TSPAN-15, PDB ID: 7RDB). Overlays were generated in Photoshop by aligning the first N-terminal helix (H1), as this region remained unchanged between the WT and mutants. Movies were generated using screen recording and Canva for final editing.

### Statistics

All statistical analysis was performed using Graph Pad Prism (San Diego, CA, United States, Version 7.05). For testing involving multiple groups one-way or two-way ANOVA was used followed by Tukey–Kramer’s post hoc testing. Summary of sample sizes for each quantitative experiment can be found in Supplementary Table 3. Immunofluorescence or immunoprecipitation experiments were repeated at least three time to ensure reproducibility using retinas and retinal sections from different animals.

### Supplementary Information

Below is the link to the electronic supplementary material.

Supplementary file1 (DOCX 3461 KB)

## Data Availability

The data generated for the current manuscript are available from the corresponding authors upon formal request.
