# Supplementary material for: Comparative study of PRPH2 D2 loop mutants reveals divergent disease mechanism in rods and cones
Source: Cell Mol Life Sci. 2023 Jul 19;80(8):214. doi: 10.1007/s00018-023-04851-3 (PMC10356684; doi:10.1007/s00018-023-04851-3)
Supplement: Supplementary file 1 — Supplementary file1 (DOCX 3461 KB) [file 18_2023_4851_MOESM1_ESM.docx]

**Supplemental Information**

**Comparative Study of PRPH2 D2 Loop Mutants Reveals Divergent Disease Mechanism in Rods and Cones**

Larissa Ikelle^#1^, Mustafa Makia^#1^, Tylor Lewis^2^, Ryan Crane^1^, Mashal Kakakhel^1^, Shannon M. Conley^3^, James Birtley^4^, Vadim Y. Arshavsky^2^, Muayyad R. Al-Ubaidi^1*^ and Muna I. Naash^1*^

^1^Department of Biomedical Engineering, University of Houston, Houston, TX, USA; ^2^Department of Ophthalmology, Duke University Medical Center, Durham, NC, USA; ^3^Department of Cell Biology, University of Oklahoma Health Sciences Center, Oklahoma City, Oklahoma, 73104, USA

^4^Epsilogen Ltd, Hammersmith, London, W6 9RH, UK

*To whom correspondence should be addressed at: Muna I. Naash (ORCID: 0000-0002-6534-5144), [mnaash@central.uh.edu](mailto:mnaash@central.uh.edu); Phone: 713-743-1651 and Muayyad R. Al-Ubaidi, (ORCID 0000-0002-4914-350X), [malubaid@central.uh.edu](mailto:malubaid@central.uh.edu); Phone: 713-743-1648; Department of Biomedical Engineering, University of Houston, 3517 Cullen Blvd. Room 2027, Houston, TX 77204-5060.

#These authors made equal contributions

Abbreviated title: Mutations in PRPH2 D2 Loop confer cell type specific effects

**Keywords:** Peripherin-2, tetraspanin, ROM1, retinal degeneration, retinitis pigmentosa, pattern dystrophy

Conflict of interest statement:

The authors declare no competing financial interests.

Number of pages: 61

Number of figures: 11 main figures and 9 supplemental figures

Abstract: 209 words

Introduction: 664 words

Discussion: 1,914 words

**
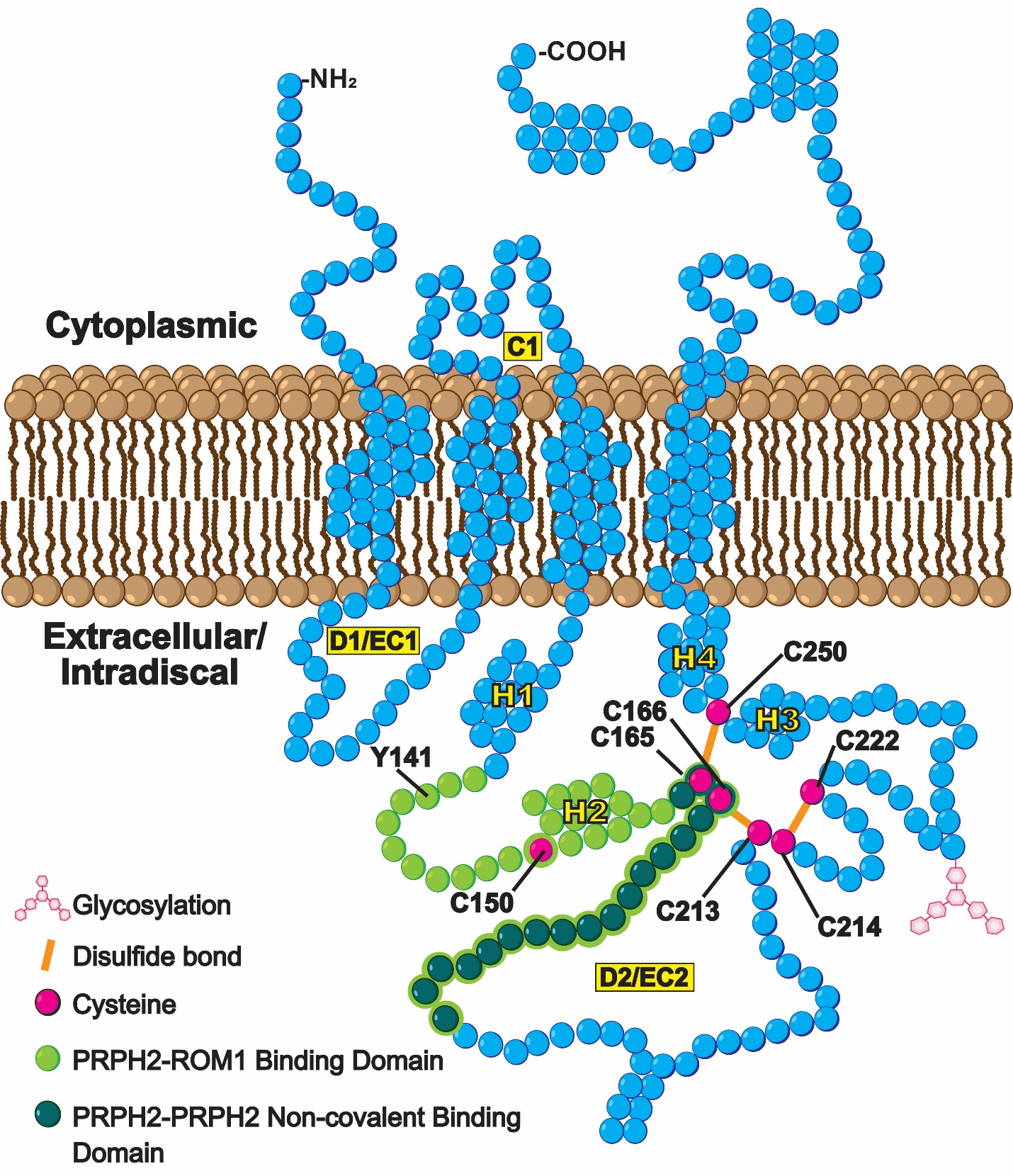
**

**Figure S1. Illustration of PRPH2 molecular structure.** Schematic of PRPH2 protein highlights its structural characteristic of 4 membrane-spanning regions, a small and a large extracellular loops (D1/EC1 and D2/EC2, respectively), and critical cysteine residues in the D2 loop, potential disulfide bonding, as well as PRPH2-PRPH2 and PRPH2-ROM1 binding domains. (H1-H4 refer to the 4 helices found in the D2 loop).

|  |  |
| --- | --- |
| **Residue** | **ASA, Å^2^** |
| Tyr 141 | 4.98 |
| Ser 150 | 66.55 |
| Cys 213 | 0.00 |
|  |  |

**Supplementary Table 1. Accessible surface area of Y141, S150 and C213.** Amino acid sidechains using the PRPH2/ROM1 cryo-EM structure was analysed using the PDBePISA server. The accessible surface areas (ASA) are given in Å^2^. Accessible surface area is reported for Ser 150 since this was the in the original crystal structure as defined in [1].


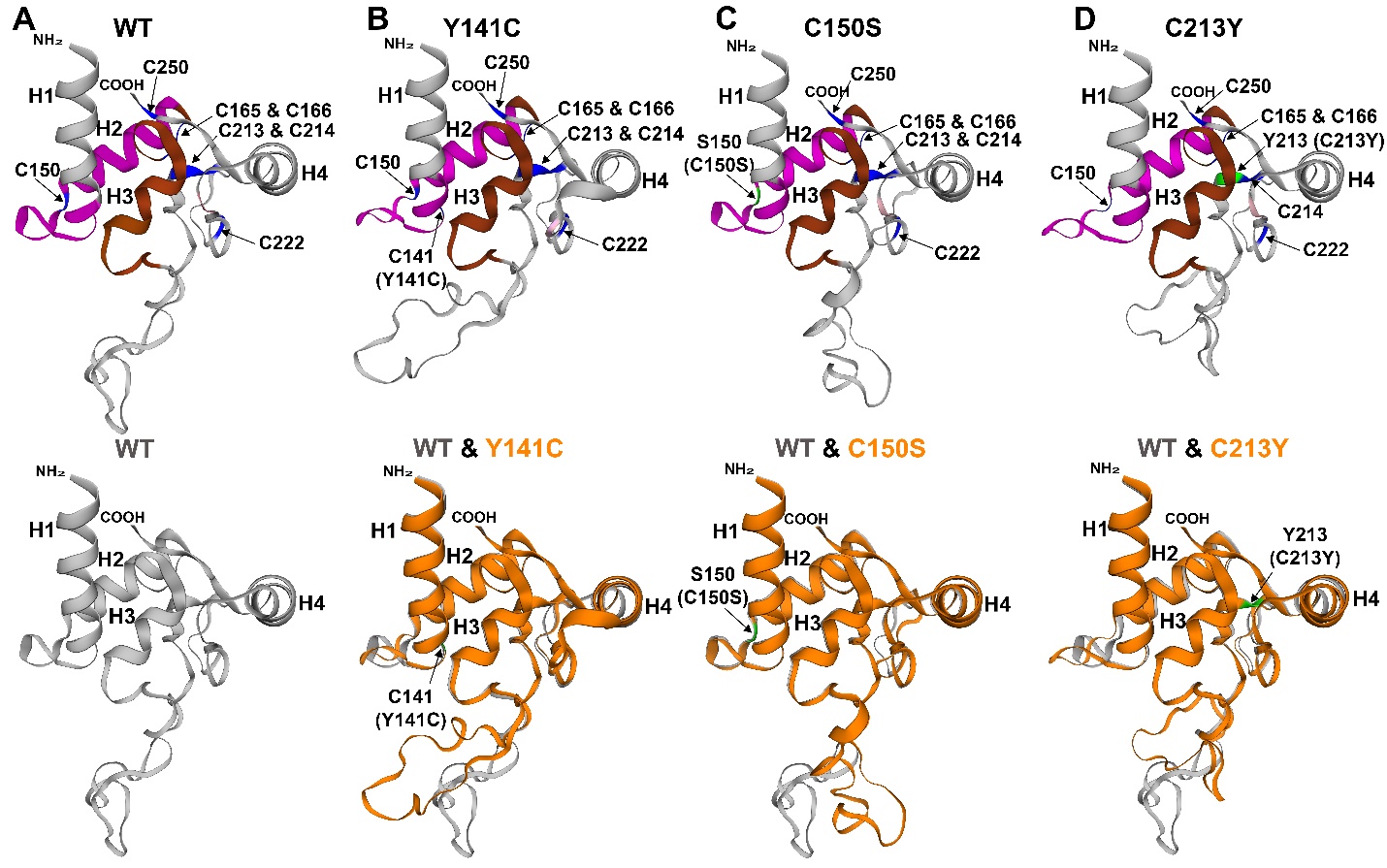


**Figure S2. Homology-modeling of *Prph2* mutants demonstrate conformational changes.** The D2 loop of PRPH2 was input into the SWISS-MODEL homology-modeling server to generate predicted structures of the D2 loop for the WT (**A**) and mutant proteins (**B-D**). The large extracellular loop of Tetraspanin-15 (Tspan-15 LEL, PDB ID: 7RDB) was used as a template. WT and mutant D2 loops were annotated with relevant binding domains. The ROM1/PRPH2 binding domain spans the regions marked in purple and brown, while the PRPH2/PRPH2 binding domain is marked in brown. Blue and pink in upper images are to mark cysteines and a glycosylation site, respectively. Cysteines in upper images are also denoted with arrows marking their specific positions. Lime green and arrows in lower images are to mark the mutations. Mutant proteins in lower images (orange) were overlaid onto the WT structure (gray) to demonstrate conformational changes.

**Movie 1. 3D prediction model of the WT D2 loop presented from multiple perspectives.** Black regions highlight the D2 loop cysteines, purple and brown mark ROM1/PRPH2 and PRPH2/PRPH2 binding domains, respectively.

**Movie 2. 3D prediction model of Y141C D2 loop presented from multiple perspectives.** Black regions highlight the D2 loop cysteines, lime green denotes the *Y141C* mutation, and purple and brown mark ROM1/PRPH2 and PRPH2/PRPH2 binding domains, respectively.

**Movie 3. 3D prediction model of C150S D2 loop presented from multiple perspectives.** Black regions highlight the D2 loop cysteines, lime green denotes the *C150S* mutation, and purple and brown mark ROM1/PRPH2 and PRPH2/PRPH2 binding domains, respectively.

**Movie 4. 3D prediction model of C213Y D2 loop presented from multiple perspectives.** Black regions highlight the D2 loop cysteines, lime green denotes the *C213Y* mutation, and purple and brown mark ROM1/PRPH2 and PRPH2/PRPH2 binding domains, respectively.

**
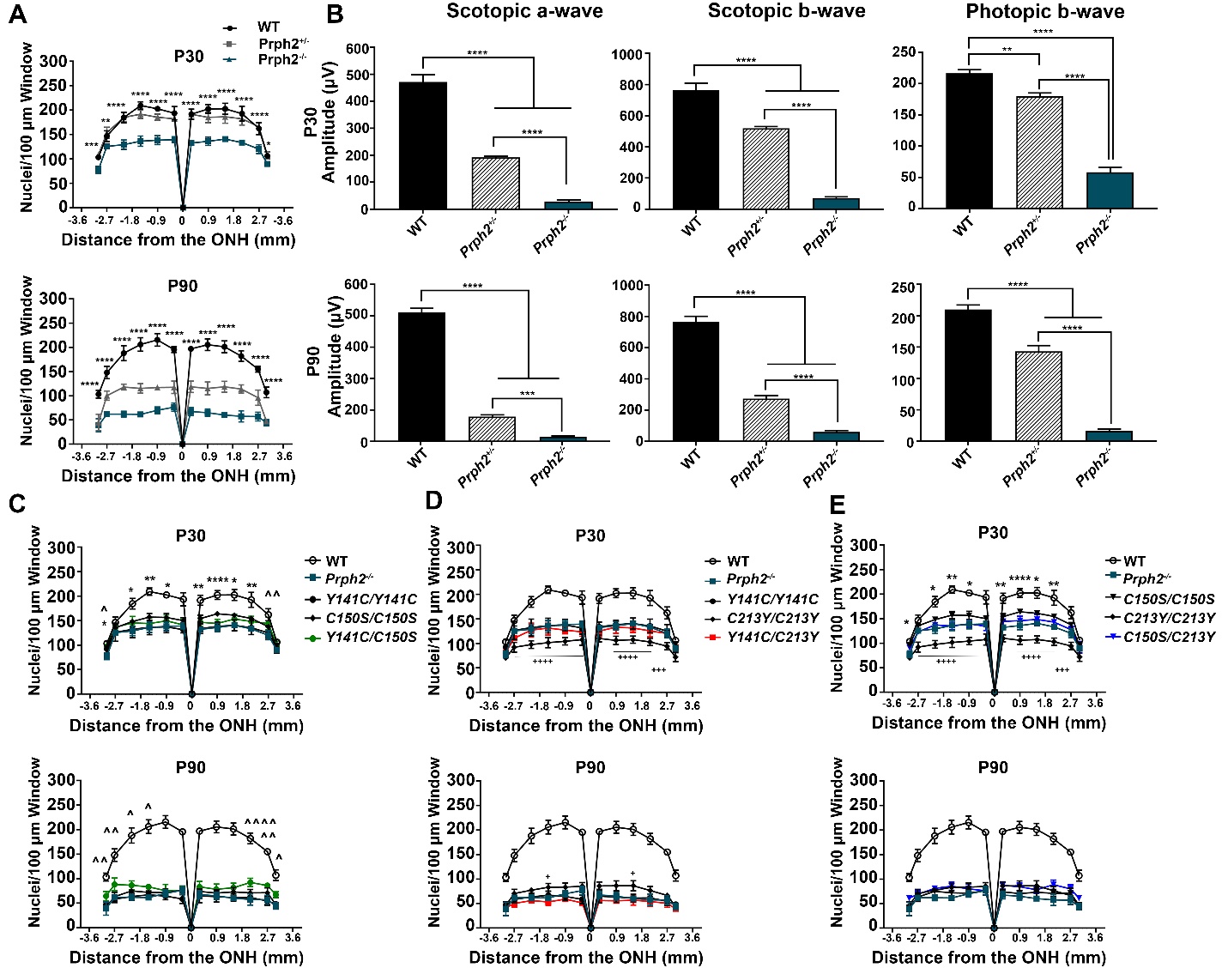
**

**Figure S3. ERG and morphometric analysis.** Full field scotopic and photopic ERGs (**B**) and morphometric analysis (**A, C-E)** were performed on WT, heterozygous, and null animals at P30 and P90. Amplitudes of scotopic a-, b-, and photopic-b-wave are presented as the mean ± SEM. N = 7 to 8. Shown in **A, C-E** are spidergrams in which ONL thickness from homozygous and compound mutant retinas (from Figure 1, main text) is plotted with WT and *Prph2^-/-^*. For ERGs (**B**) statistical analyses are from one-way ANOVA followed by Tukey-Kramer’s post hoc analysis. For morphometric data (**A, C-E**) statistical analyses are from two-way ANOVA followed by Tukey-Kramers’s post hoc analysis. Asterisks (*) in A and C indicate comparisons between the *Prph2*^-/-^ and WT retina. Asterisks (*) in E indicate comparison between the *Prph2^-/-^* and *C150S/C150S*, the circumflexes (^) in C indicate comparisons between *Y141C/C150S* and *Prph2^-/-^* and pluses (+) in D and E indicate comparisons between *C213Y/C213Y* and *Prph2^-/-^*. * is *P≤0.05*, ** is *P≤0.01*, *** is *P≤0.001,* and **** is *P≤ 0.0001.*


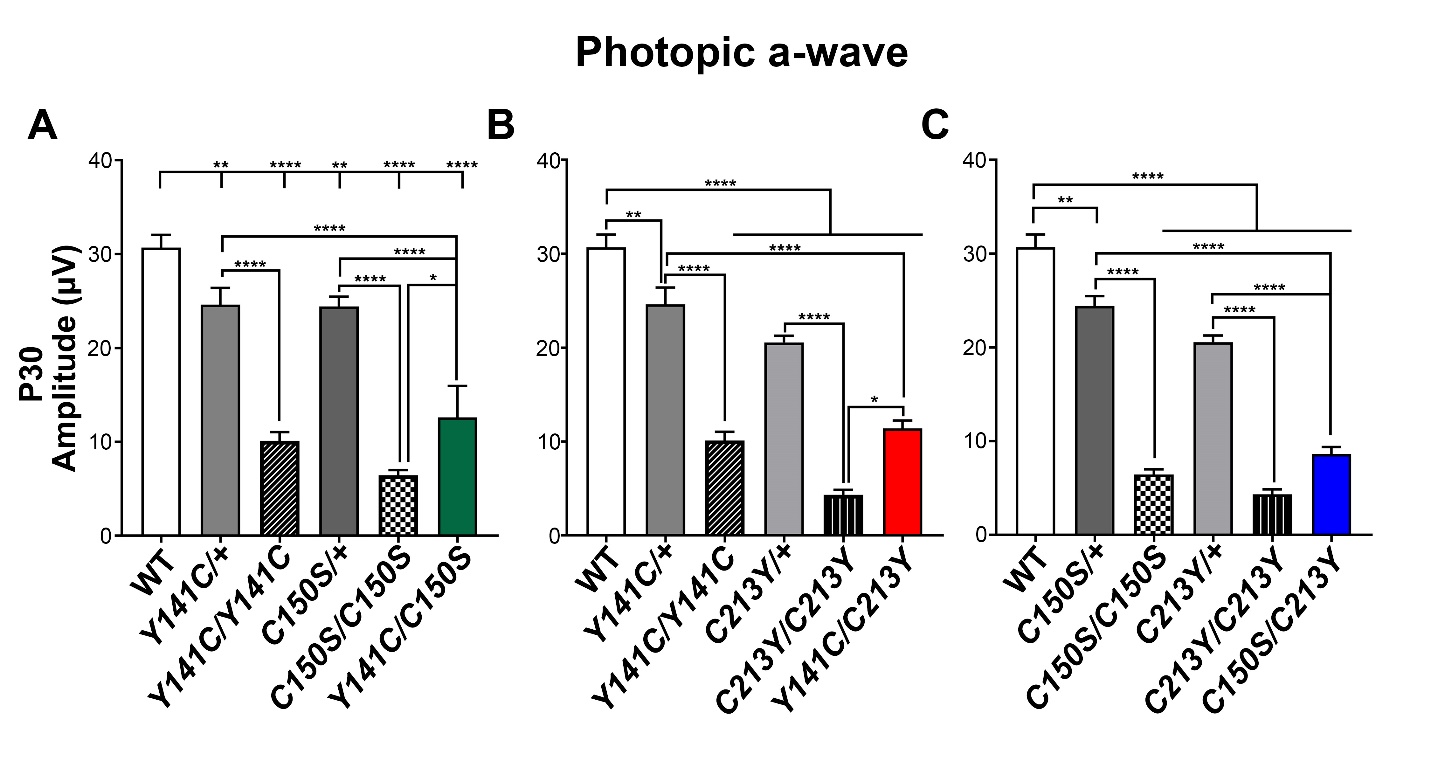


**Figure S4. Photopic a-wave of P30 animals. A-C** Full-field photopic a-wave amplitudes are presented as the mean ± SEM. White, grey, and black and white patterns bars, represent values for WT, heterozygous and homozygous, respectively. Colored bars represent the values of responses from compound animals exclusively. N = 7 to 13 animals per group. One-way ANOVA followed by Tukey-Kramer’s post-hoc testing was used to determine statistical significance. All significant pairwise comparisons are marked for: 1) pairs that share at least one allele (e.g., *C150S/+* vs. *C150S/C150S* or *C150S/Y141C*), 2) for homozygous animals and their associated compound lines (e.g., *Y141C/Y141C* vs. *C150S/C150S* and *Y141C/Y141C* vs. *Y141C/C150S*), and 3) for pairwise comparisons with WT. Other pairwise comparisons are not marked as they are not scientifically meaningful (e.g., *C150S/+* vs. *Y141C/C213Y*). * is *P≤0.05*, ** is *P≤0.01*, *** is *P≤0.001*, **** is *P≤ 0.0001.*


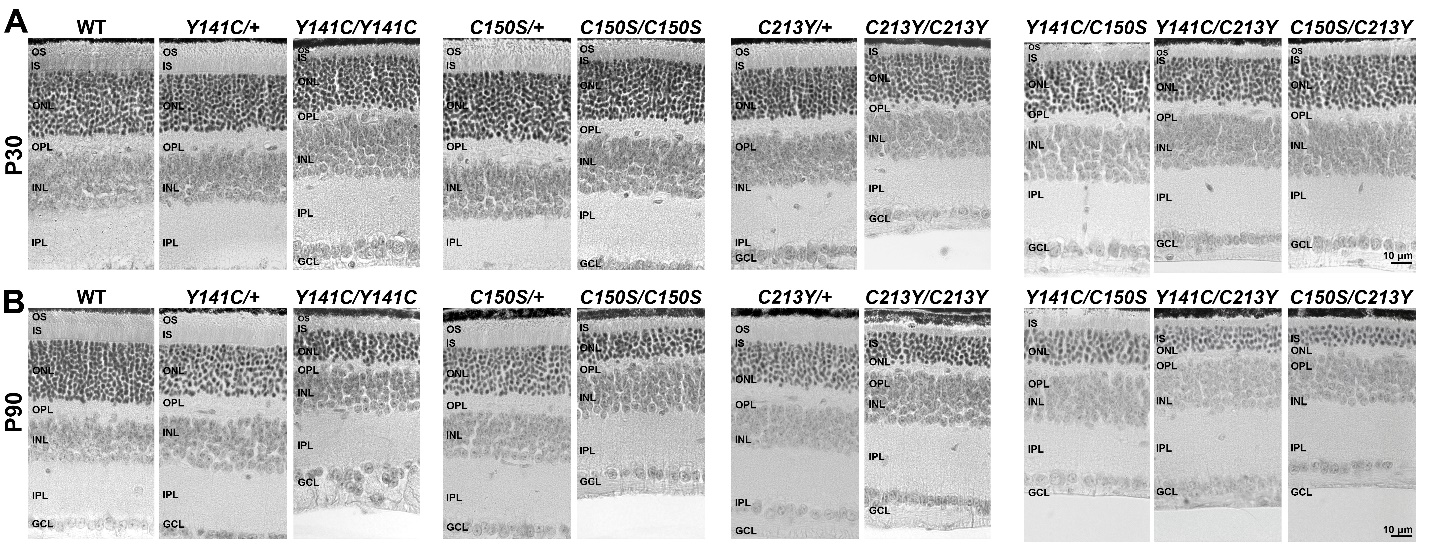


**Figure S5. Histology of compound mutants and corresponding heterozygotes and homozygotes.** Retinal cross sections of WT, homozygous, heterozygous, and compound mutants were captured at 40X, 300 µm from the optic nerve. Images were captured from P30 (**A**) and P90 (**B**) retinas. Scale bar is 10 µm. OS: outer segment, IS: inner segment, ONL: outer nuclear layer, and OPL: outer plexiform layer, INL: inner nuclear layer, IPL: inner plexiform layer, GCL: ganglion cell layer.


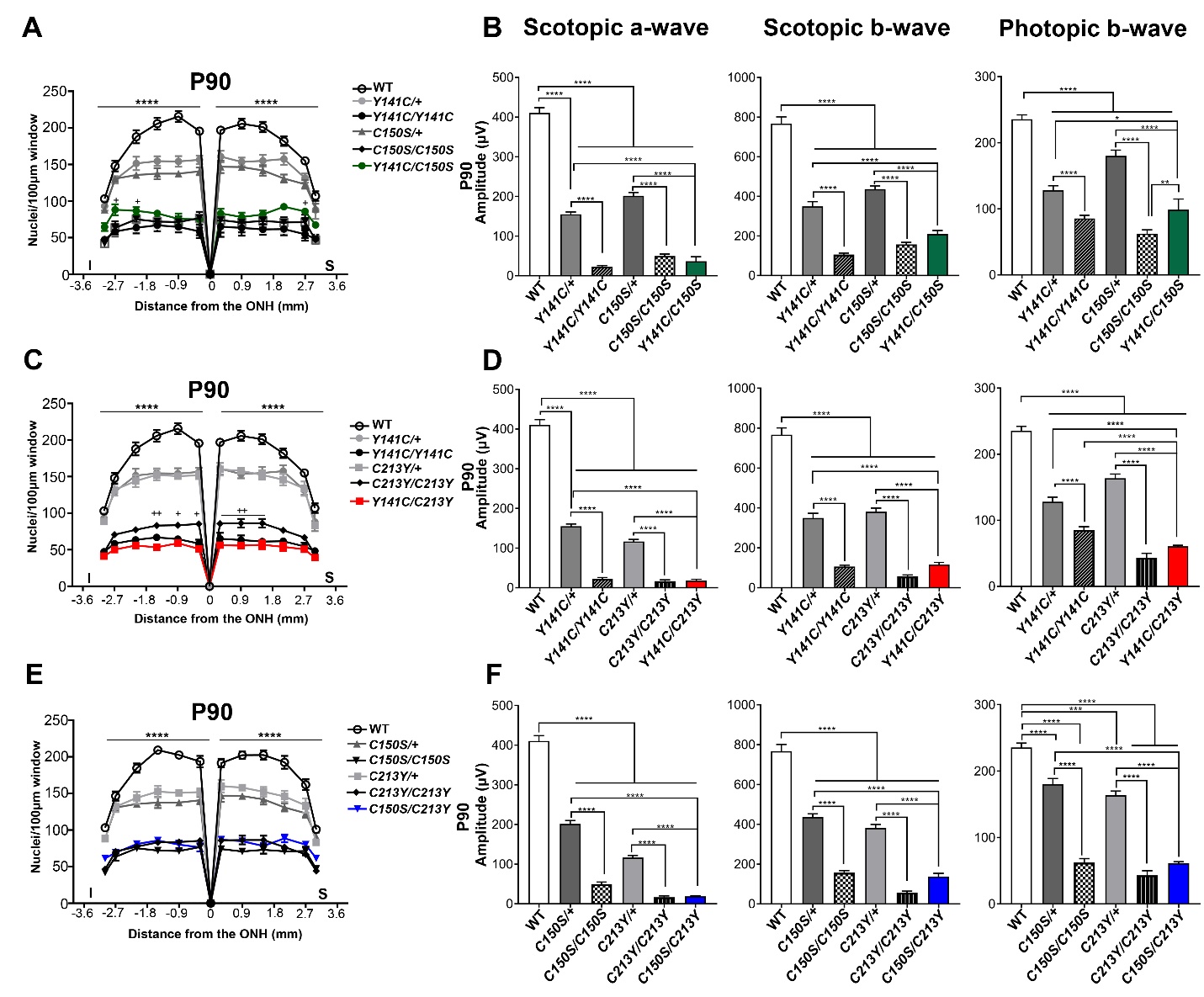


**Figure S6. Morphometry and ERG of P90 compound mutants and corresponding heterozygotes and homozygotes. A,C,E.** Morphometric analyses of H&E stained images were performed on retinal sections of the indicated genotypes at P90. Retinas were cut along the superior-inferior axis, and nuclei in the outer nuclear layer were counted in 100 µm windows. **B, D, F.** Full-field ERGs were recorded and scotopic a-, b-, and photopic-b-wave amplitudes are presented as the mean ± SEM. White, grey, and black and white patterns bars, represent values for WT, heterozygous and homozygous, respectively. Colored bars represent the values of responses from compound animals exclusively. N=7 to 13 animals per group. One-way ANOVA (ERG) or two-way ANOVA (morphometry) followed by Tukey-Kramer’s post-hoc testing was used to determine statistical significance. For morphometric data, * indicate comparisons between WT and the compound mutant (A, C, E), + indicated comparison between the compound mutant and *Y141C/Y141C* (C) or between the compound and *C213Y* (E). All significant pairwise comparisons are marked for: 1) pairs that share at least one allele (e.g., *C150S/+* vs. *C150S/Y141C*), 2) for homozygous animals and their associated compound lines (e.g., *Y141C/Y141C* vs. *C150S/C150S* and *Y141C/Y141C* vs. *Y141C/C150S*), and 3) for pairwise comparisons with WT. Other pairwise comparisons are not marked as they are not scientifically meaningful (e.g., *C150S/+* vs. *Y141C/C213Y*). * is *P≤0.05*, ** is *P≤0.01*, *** is *P≤0.001*, **** is *P≤ 0.0001.*


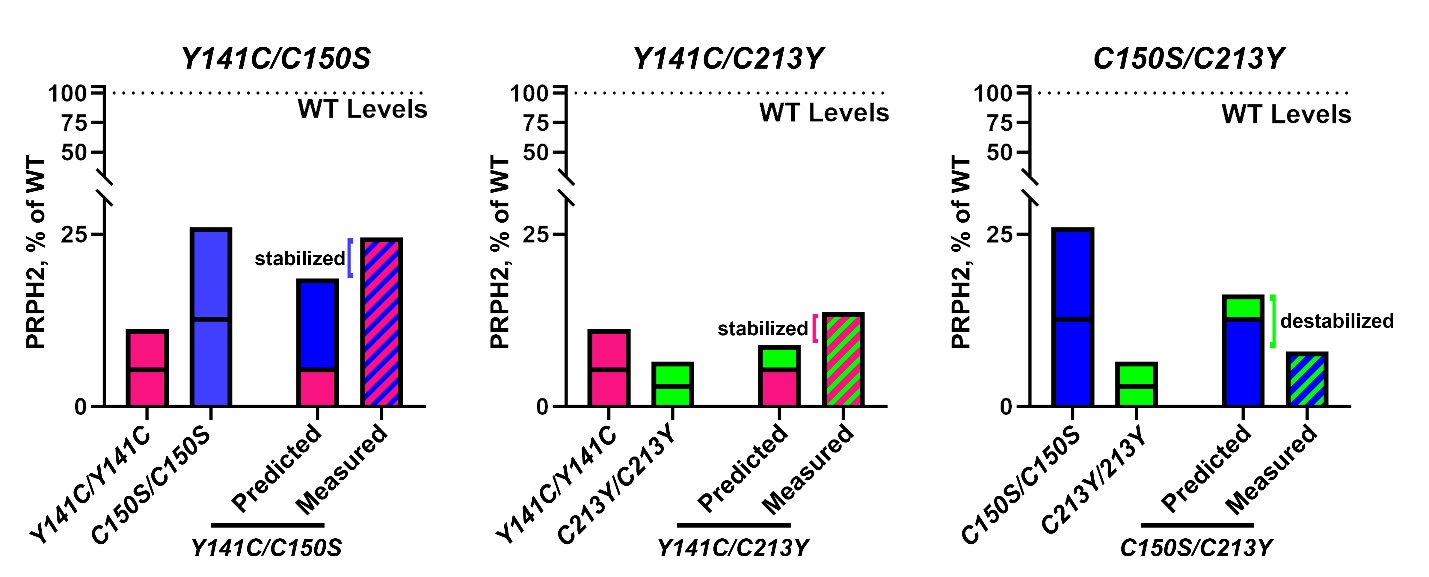


**Figure S7. Plot of predicted vs measured protein levels of PRPH2 in compound mutants.** Relative protein levels from homozygous retinas (from Fig 4. B, D, and F) are plotted as % of WT. A black line in first two bars reflects the estimated contribution of each allele. The third bar represents a predicted value for the compound mutant reflecting the amount of protein produced by each of the alleles. The fourth bar is the measured value for each compound mutant (Fig. 4 B, D, and F).

| **Statistical Analysis of PRPH2 Oligomers** | | |
| --- | --- | --- |
| **Pairwise Comparison** | **Adjusted P-Value** | **Summary** |
| **Monomer** | | |
| WT vs. *C150S/C150S* | <0.0001 | **** |
| WT vs. *C213Y/C213Y* | <0.0001 | **** |
| WT vs. *Y141C/C150S* | 0.0058 | ** |
| *Y141C+/-* vs. *Y141C/Y141C* | 0.0338 | * |
| *C150S/+* vs. *C150S/C150S* | <0.0001 | **** |
| *C213Y*+/- vs. *C213Y/C213Y* | <0.0001 | **** |
| *Y141C+/-* vs. *Y141C/C150S* | <0.0001 | **** |
| *C150S/C150S* vs. *Y141C/C150S* | <0.0001 | **** |
| *C213Y* vs. *Y141C/C213Y* | 0.0251 | * |
| **Dimer** | | |
| WT vs. *Y141C/Y141C* | <0.0001 | **** |
| WT vs. *Y141C/C150S* | <0.0001 | **** |
| WT vs. *C150S/C150S* | <0.0001 | **** |
| *Y141C+/-* vs. *Y141C/Y141C* | <0.0001 | **** |
| *C150S/+* vs. *C150S/C150S* | <0.0001 | **** |
| *Y141C+/-* vs. *Y141C/C150S* | <0.0001 | **** |
| WT vs. *C213Y/C213Y* | 0.0125 | * |
| WT vs. *Y141C/C213Y* | <0.0001 | **** |
| *C213Y+/-* vs. C213Y/C213Y | 0.0027 | ** |
| *Y141C+/-* vs. *Y141C/C213Y* | <0.0001 | **** |
| *C213Y+/-* vs. *Y141C/C213Y* | <0.0001 | **** |
| *C150S/+* vs. *C150S/C213Y* | <0.0001 | **** |
| *C213Y+/-* vs. *C150S/C213Y* | <0.0001 | **** |
| *C213Y/C213Y* vs. *C150S/C213Y* | <0.0001 | **** |
| **Abnormal High Molecular Weight Oligomer** | | |
| *Y141C+/-* vs. *Y141C/C213Y* | 0.0013 | ** |

**Supplementary Table 2. Statistical comparisons of PRPH2 oligomers.** Two-way ANOVA followed by Tukey-Kramer’s post-hoc analysis was used to determine statistical significance between monomer, dimer, and abnormal high molecular weight oligomers presented in Figure 5. Only scientifically relevant comparison is presented. ROM1 oligomers showed no statistically significant difference between all genotypes and were consequently not presented. * is *P≤0.05*, ** is *P≤0.01*, *** is *P≤0.001*, **** is *P≤ 0.0001.*


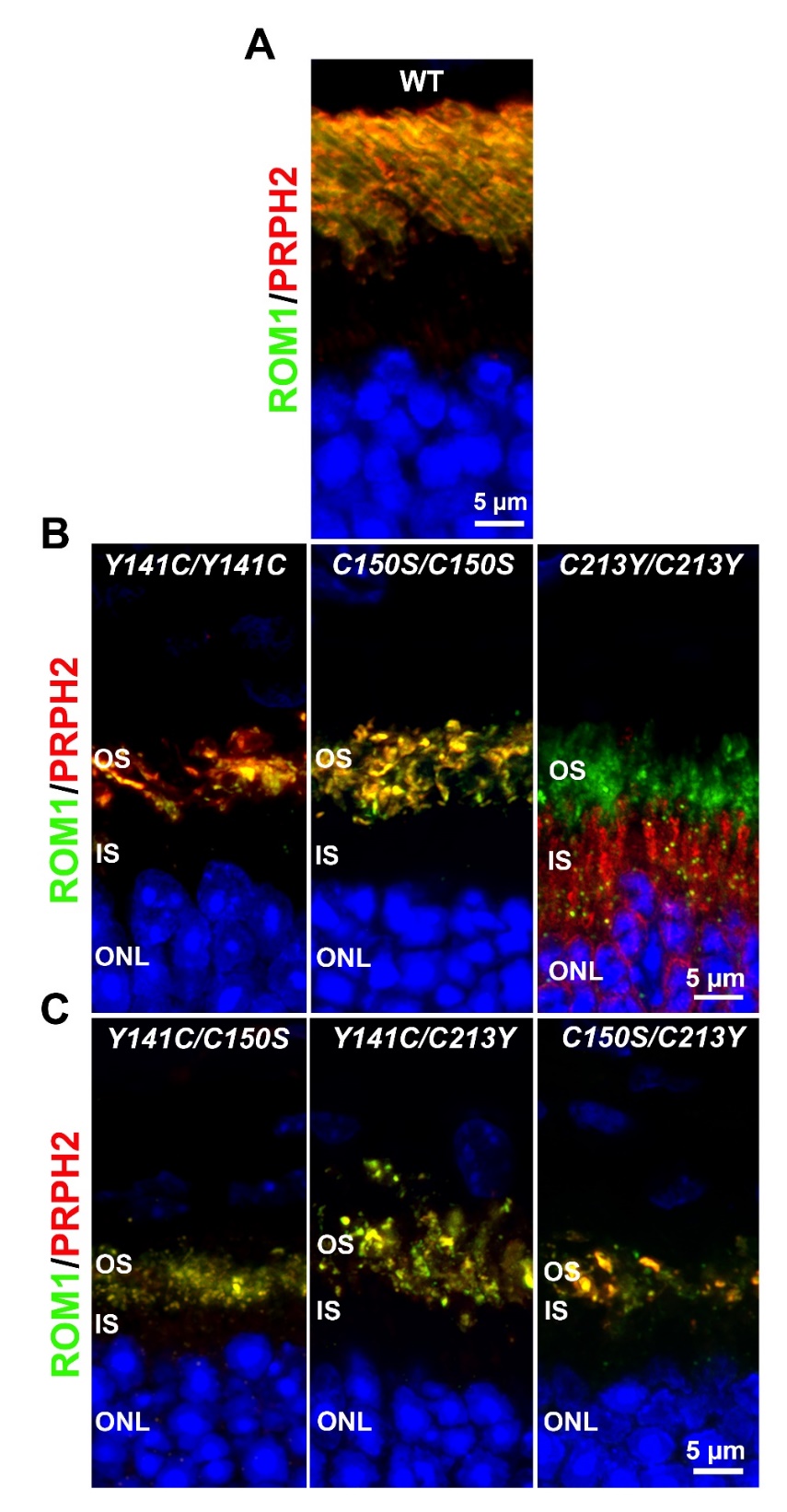


**Figure S8. Localization of PRPH2 and ROM1 in mutant retinas.** P30 retinal cross-sections from WT (**A**), homozygous (**B**) and compound mutants (**C**) were labeled for PRPH2 (red) and ROM1 (green). Images were captured at 63X magnification with 2.1X zoom. Scale bar 5 µm. OS: outer segment, IS: inner segment, and ONL: outer nuclear layer.


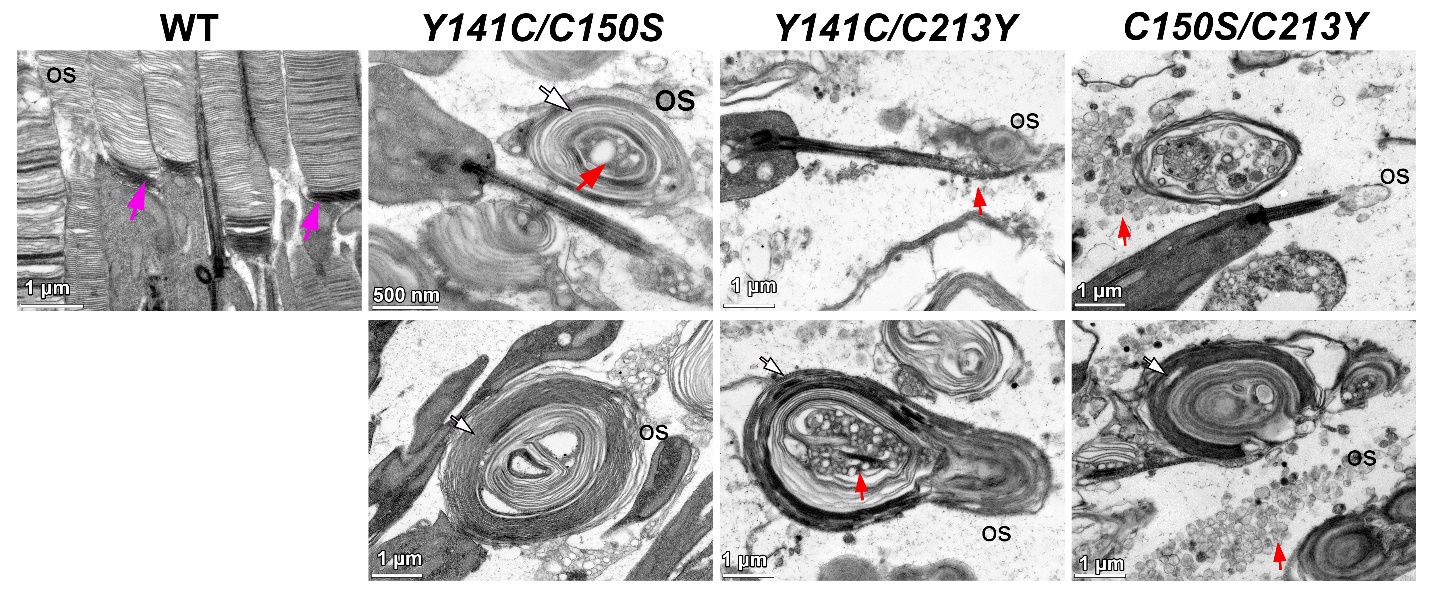


**Figure S9. Ultrastructure of P30 retinas of WT and compound mutants.** Transmission electron microscopy (TEM) on tannic acid/uranyl acetate-stained retinal sections was used to evaluate outer segment ultrastructure at P30. Arrows point to important abnormal features of mutant retinas: magenta arrows: open/nascent discs (tannic acid stained), white arrows: whorls, red arrows: ectosomes/entrapped vesicles. Scale bars are labeled in each image.

| **WT & Heterozygous Samples** | | |
| --- | --- | --- |
| **Genotype** | **Experiment** | **Sample Size** |
| WT | Immunobloting (reducing) | 6 |
|  | ERG | 13 |
|  | Morphometry | 3 |
|  | Immunobloting (non-reducing) | 6 |
|  | Cone Count | 3 |
| Y141C/+ | Immunobloting (reducing) | 5 |
|  | ERG | 12 |
|  | Morphometry | 3 |
|  | Immunobloting (non-reducing) | 6 |
|  | Cone Count | 3 |
| C150S/+ | Immunobloting (reducing) | 5 |
|  | ERG | 8 |
|  | Morphometry | 3 |
|  | Immunobloting (non-reducing) | 6 |
|  | Cone Count | 3 |
| C213Y/+ | Immunobloting (reducing) | 6 |
|  | ERG | 12 |
|  | Morphometry | 3 |
|  | Immunobloting (non-reducing) | 6 |
|  | Cone Count | 3 |
|  | | |
| **Homozygous Samples** | | |
| **Genotype** | **Experiment** | **Sample Size** |
| Y141C/Y141C | Immunobloting (reducing) | 6 |
|  | ERG | 12 |
|  | Morphometry | 3 |
|  | Immunobloting (non-reducing) | 9 |
|  | Cone Count | 3 |
| C150S/C150S | Immunobloting (reducing) | 6 |
|  | ERG | 8 |
|  | Morphometry | 3 |
|  | Immunobloting (non-reducing) | 6 |
|  | Cone Count | 3 |
| C213Y/C213Y | Immunobloting (reducing) | 6 |
|  | ERG | 9 |
|  | Morphometry | 3 |
|  | Immunobloting (non-reducing) | 4 |
|  | Cone Count | 3 |
|  | | |
| **Compound Mutant Samples** | | |
| **Genotype** | **Experiment** | **Sample Size** |
| Y141C/C150S | Immunobloting (reducing) | 5 |
|  | ERG | 7 |
|  | Morphometry | 3 |
|  | Immunobloting (non-reducing) | 5 |
|  | Cone Count | 3 |
| Y141C/C213Y | Immunobloting (reducing) | 5 |
|  | ERG | 13 |
|  | Morphometry | 3 |
|  | Immunobloting (non-reducing) | 6 |
|  | Cone Count | 3 |
| C150S/C213Y | Immunobloting (reducing) | 5 |
|  | ERG | 7 |
|  | Morphometry | 3 |
|  | Immunobloting (non-reducing) | 6 |
|  | Cone Count | 3 |
|  | | |
| **Experiment** | **Statistical Test** | **Post hoc testing** |
| Immunobloting (reducing) | One-Way ANOVA | Tukey-Kramer |
| ERG | One-Way ANOVA | Tukey-Kramer |
| Morphometry | Two-Way ANOVA | Tukey-Kramer |
| Immunobloting (non-reducing) | Two-Way ANOVA | Tukey-Kramer |
| Cone Count | One-Way ANOVA | Tukey-Kramer |

**Supplementary Table 3. Sample sizes and statistical testing used in this study.**

**References**

1. El Mazouni D and Gros P (2022) Cryo-EM structures of peripherin-2 and ROM1 suggest multiple roles in photoreceptor membrane morphogenesis. Science Advances 8:eadd3677. <https://doi.org/doi:10.1126/sciadv.add3677>
